# The Smc5/6 complex is a DNA loop-extruding motor

**DOI:** 10.1038/s41586-023-05963-3

**Published:** 2023-04-19

**Authors:** Biswajit Pradhan, Takaharu Kanno, Miki Umeda Igarashi, Mun Siong Loke, Martin Dieter Baaske, Jan Siu Kei Wong, Kristian Jeppsson, Camilla Björkegren, Eugene Kim

**Affiliations:** 1 Max Planck Institute of Biophysics, Frankfurt am Main, Germany; 2 Department of Cell and Molecular Biology, Karolinska Institutet, Stockholm, Sweden; 3 Department of Biosciences and Nutrition, Karolinska Institutet, Huddinge, Sweden

**Keywords:** Single-molecule biophysics, Chromosomes, Single-molecule biophysics

## Abstract

Structural maintenance of chromosomes (SMC) protein complexes are essential for the spatial organization of chromosomes^1^. Whereas cohesin and condensin organize chromosomes by extrusion of DNA loops, the molecular functions of the third eukaryotic SMC complex, Smc5/6, remain largely unknown^2^. Using single-molecule imaging, we show that Smc5/6 forms DNA loops by extrusion. Upon ATP hydrolysis, Smc5/6 reels DNA symmetrically into loops at a force-dependent rate of one kilobase pair per second. Smc5/6 extrudes loops in the form of dimers, whereas monomeric Smc5/6 unidirectionally translocates along DNA. We also find that the subunits Nse5 and Nse6 (Nse5/6) act as negative regulators of loop extrusion. Nse5/6 inhibits loop-extrusion initiation by hindering Smc5/6 dimerization but has no influence on ongoing loop extrusion. Our findings reveal functions of Smc5/6 at the molecular level and establish DNA loop extrusion as a conserved mechanism among eukaryotic SMC complexes.

## Abstract (web summary)

Using single-molecule imaging, the authors show that Smc5/6 forms DNA loops by extrusion, which establishes DNA loop extrusion as a conserved mechanism among eukaryotic SMC complexes.

## Main

The structural maintenance of chromosomes (SMC) complexes, such as condensin, cohesin and the Smc5/6 complex, control chromosome organization and regulate most genomic processes, including gene expression, chromosome segregation and DNA repair^1^. These multisubunit complexes are composed of a characteristic ring-shaped trimeric structure containing a pair of SMC ATPases and a kleisin protein, as well as additional regulatory subunits^3^. Cohesin folds interphase chromosomes into chromatin loops and topologically associated domains^4–7^, whereas condensin organizes mitotic chromosomes in the form of hierarchically nested loops^8,9^. Single-molecule experiments have shown that condensin and cohesin form DNA loops by an active extrusion process^10–12^. However, whether loop extrusion is a conserved feature of all SMC complexes or specific to condensin and cohesin remains an open question.

Unlike condensin and cohesin, the functions of the third eukaryotic SMC complex, Smc5/6, are considerably less explored. Smc5/6 has been implicated in repair of DNA damage by homologous recombination^13,14^, in the promotion of chromosome segregation^15,16^ and in replication fork stability and progression^17,18^. At the molecular level different modes of action have been suggested, including DNA–DNA tethering^17,19^, DNA compaction through direct interactions between multiple complexes^20^ and efficient recognition and stabilization of supercoiled and catenated DNA^15,20,21^. In regard to its structural similarities with condensin and cohesin, it seems reasonable to predict that Smc5/6 also performs DNA loop extrusion and/or translocation. However, Smc5/6 also contains complex-specific features that might prevent such activities, making such a prediction more uncertain^22–24^.

**Fig. 1:**
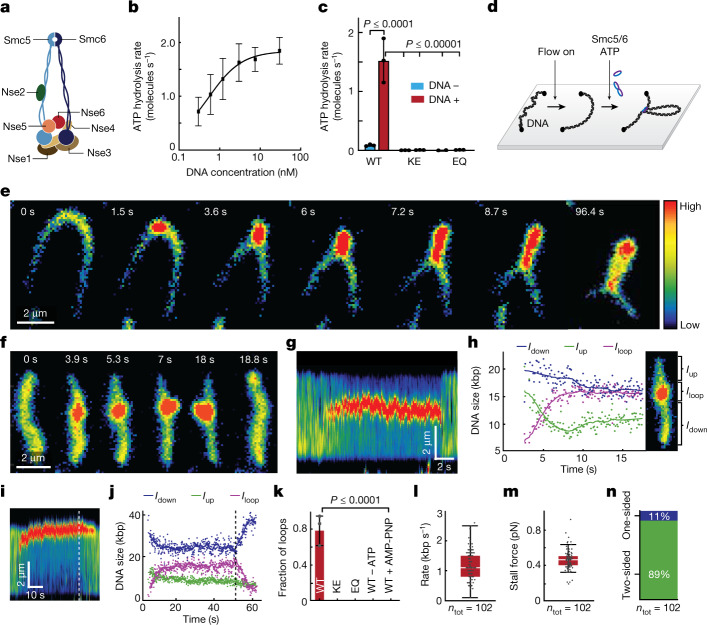
Real-time imaging of loop extrusion by Smc5/6. **a**, Cartoon of the *S. cerevisiae* Smc5/6 octameric structure. **b**, ATPase activity of the WT Smc5/6 octameric complex with different concentrations of DNA. Experimental data were fitted to a stimulatory dose–response model by nonlinear regression; mean ± s.d. from four independent measurements. **c**, ATPase activity of WT, KE and EQ Smc5/6 complexes in the absence or presence of 30 nM plasmid DNA; mean ± s.d. from three independent measurements. **d**, Schematic of DNA loop-extrusion assay. **e**, Series of images showing DNA loop-extrusion intermediates induced by Smc5/6 complex under constant buffer flow. **f**,**g**, Images (**f**) and fluorescence intensity kymograph (**g**) of a DNA molecule showing DNA loop extrusion in the absence of buffer flow. **h**, DNA lengths calculated from the kymograph in **g** for regions outside the loop (*I*_up_ and *I*_down_) and the loop region itself (*I*_loop_). **i**,**j**, Kymograph (**i**) and calculated DNA lengths (**j**) for a loop-extrusion event followed by loop release via gradual shrinkage. Dashed lines in **i**,**j** indicate the start of loop shrinkage. Data in **e**—**j** represent typical events observed more than ten times in three independent experiments. **k**, DNA loop-forming fractions (mean ± s.d.) in the presence of ATP and 2 nM Smc5/6 WT, ATPase mutant complexes as in **c** and WT in the absence of ATP or presence of AMP-PNP. *n*_tot_ = 233, 121, 93, 84 and 106, respectively. **l**,**m**, Box-and-whisker plots of Smc5/6 loop extrusion showing rates (**l**) and stalling force (**m**). *n*_tot_ = 102 molecules, median ± 1.5× interquartile rate (IQR). **n**, Fraction of loop-extrusion events exhibiting two- or one-sided DNA reeling, as determined by observation of DNA length decrease in nonloop regions (*I*_up_ and *I*_down_ in **h**,**j**). Data in **k**–**n** are from three independent experiments. Source data

**Extended Data Fig. 1:**
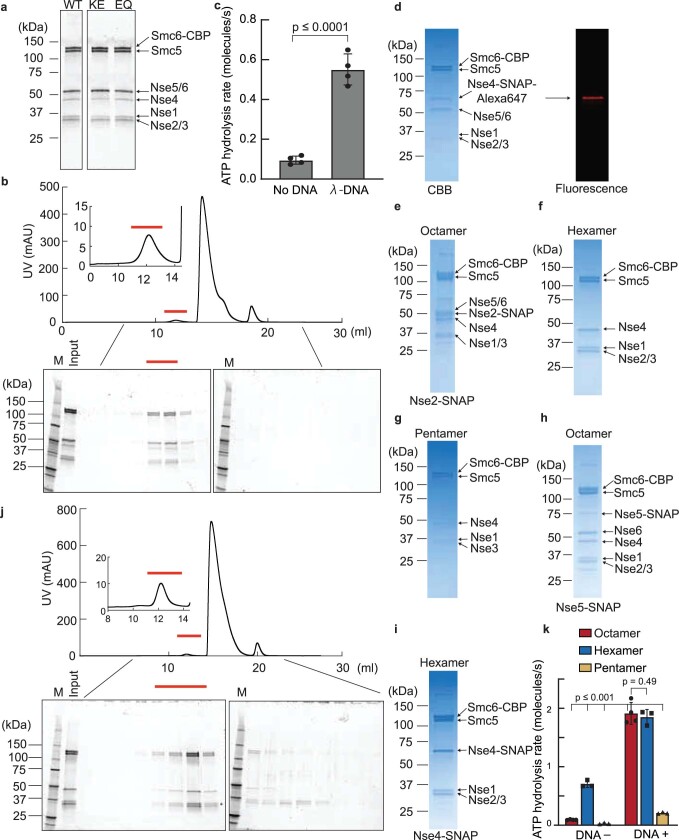
Purifications and ATPase activity of octameric and hexameric Smc5/6 complexes. (a) Oriole stained SDS-PAGE gel for purified Smc5/6 and mutants. (b) Size-exclusion chromatography (SEC) of Smc5/6 octamers. The chromatogram and Oriole-stained SDS-PAGE for each fraction are shown. Peak fractions are indicated with red bar. (c) ATP hydrolysis rate for wild type Smc5/6 with/without λ-DNA. Mean ± S.D. obtained from 4 independent measurements. (d) Fluorescently-labeled Smc5/6 with a SNAP tag on Nse4. CBB staining and fluorescence detection of SDS-PAGE are shown. (e,f,g,h,i) CBB stained SDS-PAGE for the Smc5/6 complex with a SNAP tag on Nse2 (e), Hexameric Smc5/6 complex lacking Nse5/6 (f), Pentameric Smc5/6 complex lacking Nse2/5/6 (g), Octameric Smc5/6 complex with a SNAP tag on Nse5 (h), Hexameric Smc5/6 complex lacking Nse5/6 with a SNAP tag on Nse4 (i). (j) SEC of the Smc5/6 hexamer. The chromatogram and Oriole-stained SDS-PAGE for each fraction are shown. Peak fractions are indicated with red bar. (k) ATP hydrolysis rates of octamer, hexamer, and pentamer in the absence and presence of 30 nM plasmid DNA. Mean ± S.D. obtained from 3 or 4 independent measurements. *P* values are shown; two-tailed Student’s *t*-test. Source data

**Extended Data Fig. 2:**
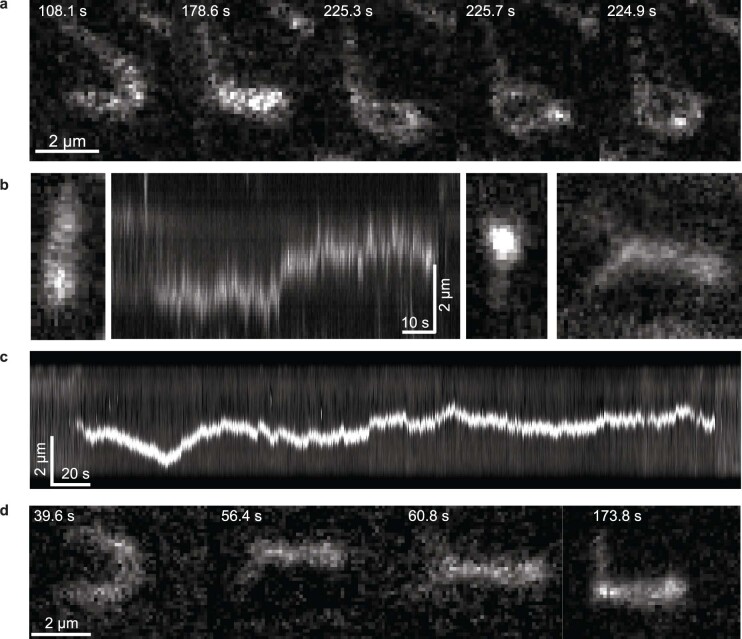
Characteristics of the Smc5/6-mediated DNA loop extrusion. (a) Snapshots showing the process of loop extrusion on a sticky surface under side flow revealing the O-shape topology of the DNA loop. (b) Snapshots (first and third panel from the left) from the first and the last frames of the kymograph (second panel) showing a loop extrusion event in the absence of flow. The fourth panel shows the same loop upon the application of side-flow confirming that the observed DNA punctum is a loop. (c) A kymograph of a DNA molecule showing a Smc5/6-mediated loop that exhibits diffusion along the DNA. (d) Snapshots showing diffusion of an extruded loop. The side-flow reveals that the stem of the loop moves along the DNA. (b,c,d) are representative of five independent experiments.

**Extended Data Fig. 3:**
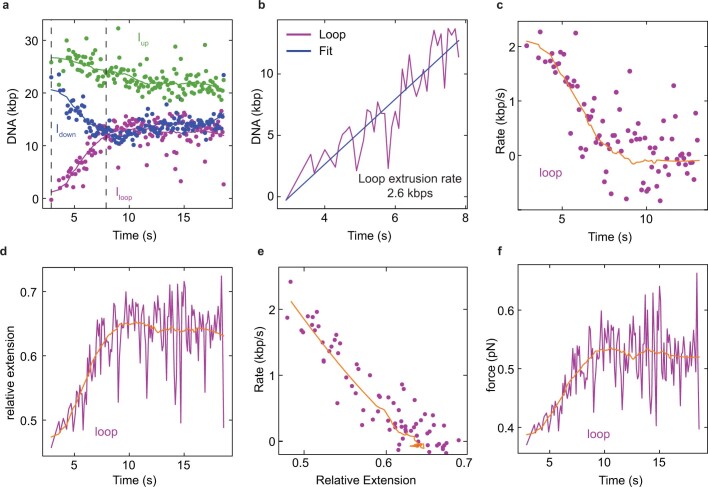
Quantification of Smc5/6-mediated loop extrusion kinetics extracted from a single looping event. (a) Kinetics of loop extrusion showing changes in DNA sizes from different sections (I_down_, I_loop_, I_up_) over time. The linear region within the two dashed lines was used to obtain the initial loop extrusion rate via linear fit as shown in (b). (c) The change of loop extrusion rate during the loop growth, which was calculated using the change in loop sizes in a moving time window of 2 s. (d) The simultaneous change in relative DNA extension as a function of time. (c,d) shows that the decrease of extrusion rate is correlated with the increase in DNA tension. (e) Scatter plot of loop extrusion rate with the relative extension, taken from (c,d), showing that above extension value of 0.6 the rate is close to zero. (f) Change in tension on the DNA with time. The force values were extracted from the values of relative DNA extension in (d) and converted to the known force–extension relation. (a–f) correspond to the single DNA loop extrusion event shown in Fig. 1f–h, and solid lines in a, c–f show running averages over 50 points. (a,b,c,d,e,f) are representative of five independent experiments.

**Extended Data Fig. 4:**
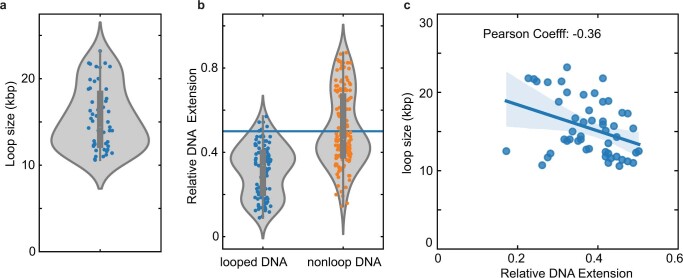
Statistics of the characteristics of Smc5/6-extruded DNA loops. (a) Loop size distribution (violin plot) from DNA looping events (N_loop_ = 100 molecules). (b) Violin plot distributions of the initial relative extension of DNA which formed loops (blue, N_tot_ = 100 molecules) or not (orange, N_tot_ = 140 molecules). Extension values were collected before the loop extrusion events started. Only 6% of the loop extrusion events (N = 100) were observed on DNA molecules which were stretched beyond an extension value of 0.6 (indicated by the horizontal line). (c) Scatter plot of loop sizes versus the relative DNA extension showing a negative correlation with Pearson coefficient of −0.36, and linear regression fit with 95 % confidence interval indicated by the shaded area. (a–c) are obtained over five independent experiments.

Here, we therefore isolated *Saccharomyces cerevisiae* Smc5/6 to examine its DNA loop-extrusion activity. Size-exclusion chromatography confirmed that the isolate contained the wild-type (WT) octameric complex with all subunits present at roughly 1:1 stoichiometry (Fig. 1a and Extended Data Fig. 1a,b; for gel source data, see Supplementary Fig. 1). The complex showed DNA-stimulated ATPase activity with a maximum rate of hydrolysis of 1.9 molecules s^–1^ (Fig. 1b and Extended Data Fig. 1c), similar to previously recorded activity ranges of Smc5/6 and other SMC complexes^10–12,20,21^. As expected, no ATP hydrolysis was detected for complexes in which both Smc5 and Smc6 were mutated to prevent ATP binding (KE mutants) or block ATP hydrolysis (EQ mutants) (Fig. 1c). We then tested the activity of Smc5/6 using a single-molecule assay that allows for direct visualization of loop extrusion mediated by SMC complexes^10,11,25^ (Fig. 1d). First, both ends of linear 48.5-kilobase-pair (kbp) λ-DNA molecules were tethered to a passivated glass surface and stained with Sytox Orange (SxO). DNA molecules were then stretched by buffer flow perpendicular to the DNA axes and imaged by total internal reflection microscopy. Following the addition of Smc5/6 and ATP under constant buffer flow, we observed that DNA was initially concentrated into one spot and then gradually extended into an elongating loop (Fig. 1e, Extended Data Fig. 2a and Supplementary Video 1). We observed loop formation on the majority of DNA molecules (78%, *n*_tot_ = 233 for 2 nM Smc5/6 and duration of 1,000 s). Looping events were also observed in the absence of buffer flow, as a loosely compacted DNA punctum increasing in size over time (Fig. 1f and Supplementary Video 2). Application of buffer flow after maturation of the DNA punctum further verified it as a single loop (Extended Data Fig. 2b). Fluorescence intensity kymographs of DNA (Fig. 1g) and the corresponding estimation of DNA length within the loop (*I*_loop_) and outside the loop (*I*_up_, *I*_down_) (Fig. 1h and Extended Data Fig. 3a) showed progressive growth of the loop (average loop size about 16 kbp, *n*_tot_ = 100; Extended Data Fig. 4a), at the expense of DNA outside of the loop, until reaching a plateau. Once extrusion was halted, the loops occasionally moved along DNA in either direction (Extended Data Fig. 2c,d) and were finally released (71%, *n*_tot_ = 202), either spontaneously in a single step (Fig. 1f,g; 39%, *n*_tot_ = 202) or by gradual shrinking of loops (Fig. 1i,j; 32%, *n*_tot_ = 202). We observed no DNA looping in the absence of ATP, in the presence of a non-hydrolysable analogue of ATP (AMP-PNP) or when the WT complex was replaced by ATP binding (KE)- or ATP hydrolysis (EQ)-deficient mutants (Fig. 1k). Together, this demonstrates that Smc5/6 can form loops in an ATP hydrolysis-dependent manner by active extrusion of DNA.

We next estimated the speed of loop extrusion (Fig. 1l; *n*_tot_ = 102) from the initial slopes of the loop growth curves (Fig. 1h and Extended Data Fig. 3b), which yielded a rate of 1.1 ± 0.5 kbp s^–1^, a value similar to those reported for human cohesin (0.5**–**1.0 kbp s^–1^)^11,12^ and yeast condensin (0.6 kbp s^–1^)^10^. Loop extrusion by Smc5/6 was force sensitive (Extended Data Fig. 3c–f), again similar to condensin and cohesin. We observed minimal loop formation (approximately 6%) when DNA was stretched above 60% of its contour length, with a corresponding force of around 0.5 pN (Extended Data Fig. 4b,c). The average stalling force, estimated from the value of relative DNA extension at which loop extrusion was halted and converted to the known force–extension relation^10^, again yielded 0.5 ± 0.1 pN (Fig. 1m). This was close to values previously reported for condensin (0.5 pN)^26^ and cohesin (below 0.8 pN)^12^. The majority of Smc5/6-mediated loop-extrusion events (89%, *n*_tot_ = 102 molecules; Fig. 1n) were ‘two-sided’ because the DNA length decreased on both sides of the loops in flow-stretched imaging (Fig. 1e), and in the estimated DNA length (*I*_up_, *I*_down_) in the absence of flow (Fig. 1h). In summary, the characteristics of loop extrusion mediated by Smc5/6 closely resemble those previously observed for both cohesin and condensin and are most similar to cohesin, which also performs two-sided extrusion.

**Fig. 2:**
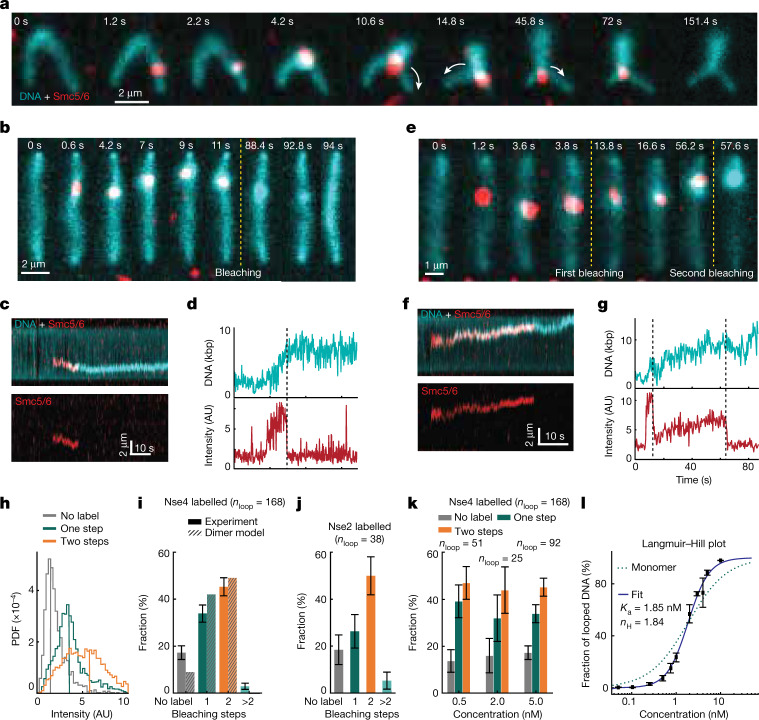
Dimers of Smc5/6 complexes extrude DNA loops. **a**,**b**,**e**, Snapshots of image overlays showing SxO-stained DNA (cyan) and Alexa 647-labelled Smc5/6 (red) during loop extrusion in the presence (**a**) and absence of buffer flow (**b**,**e**), and exhibiting one (**b**) or two photobleaching events (**e**). Arrows in **a** indicate the direction of Smc5/6 movement. **c**,**f**, Kymographs of the loop-extrusion events in **b** (**c**) and **e** (**f**) depicting overlays of DNA and Smc5/6 (top) and Smc5/6 (bottom). **d**,**g**, Time traces of DNA length (top) and Smc5/6 fluorescence intensity (bottom) determined from **c** (**d**) and **f** (**g**), with bleaching events indicated by dashed vertical lines. **h**, Probability density function (PDF) of fluorescence intensity for loop-extrusion events exhibiting either no (*n*_tot_ = 8) Alexa 647 signal or one-(*n*_tot_ = 11) or two-step (*n*_tot_ = 21) bleaching. **i**, Fraction of loop-extruding Smc5/6 events that showed either none, one, two or more bleaching steps. Dashed bars denote the calculated probabilities for finding none, one or two labels assuming that all loop-extruding complexes are dimers with labelling efficiency of 68%. **j**, Fraction of the number of bleaching steps for Nse2-labelled Smc5/6 during loop extrusion with labelling efficiency of 70%. Data in **e**—**j** represent five or more independent experiments. **k**, Histograms showing the number of bleaching steps observed during loop-extrusion events at indicated Smc5/6 concentrations. Data in **i**–**k** indicate respective fractions of total looping events (*n*_loop_) with 95% confidence interval from at least three independent experiments. **l**, Langmuir–Hill plot showing the fraction of DNA substrates that formed loops as a function of Smc5/6 concentration (solid squares); mean ± s.d. from three independent experiments. The respective fit (solid line) indicates cooperative behaviour with Hill coefficient (*n*_H_) = 1.84, deviating from the Hill–Langmuir function expected for exclusively monomeric loop extrusion (*n*_H_ = 1, dotted line). Experiments were performed using the WT octameric complex and at 1,000 s duration. AU, arbitrary units.

**Extended Data Fig. 5:**
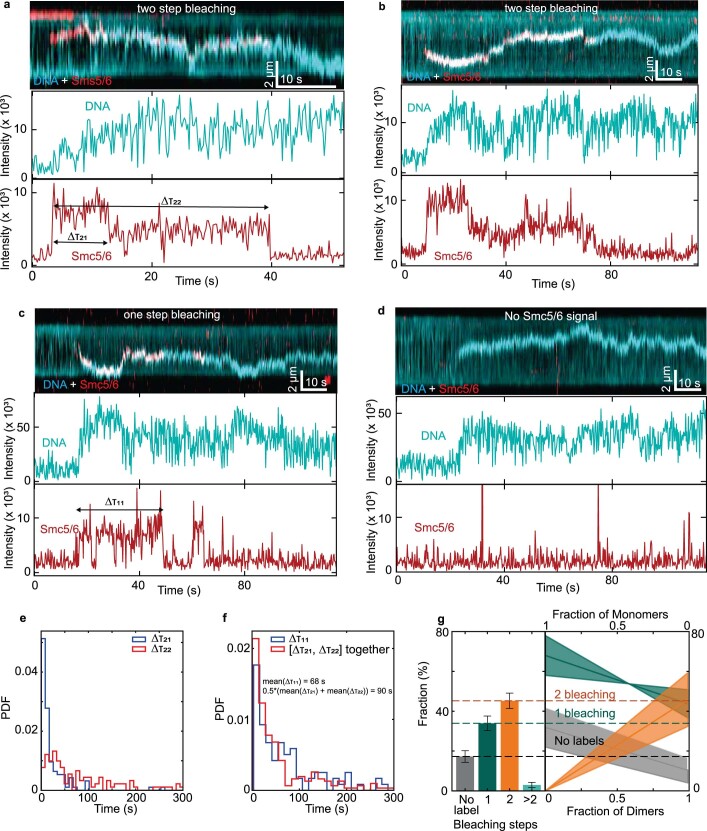
Additional examples of photobleaching events from Alexa-647 labeled Smc5/6 during DNA loop extrusion. Kymograph (top of each section) of DNA (cyan) and Smc5/6 (red). Time traces of DNA (middle of each section) and Smc5/6 fluorescence intensity (bottom of each section) determined from the corresponding kymographs showing two bleaching steps (a,b), one bleaching step (c), and no Smc5/6 signal at the loop initiation position (d). The corresponding bleaching times Δ*τ*_21_ and Δ*τ*_22_ in (a, bottom), and Δ*τ*_11_ in (b, bottom) are indicated with arrows. Intensity values displayed in Smc5/6 time-traces were extracted on the same pixel positions as the DNA puncta intensities. (e) The bleaching time distributions extracted from two bleaching step traces. (f) The bleaching time distributions Δ*τ*_11_ in a one-step bleaching traces (blue). When the two bleaching times Δ*τ*_21_, Δ*τ*_22_ from the two-step bleaching trace are put together in a histogram, they resemble the single-step distribution. Note that the average time of one-step bleaching traces (68 s) is similar to the average of the times (Δ*τ*_21_, Δ*τ*_22_) of the two-step bleaching traces (90 s). (g, left panel) Fraction of loop extruding Smc5/6 events that displayed either no, one, two or more bleaching steps as shown in Fig. 2i. Error bars with 95% confidence interval. N_loop_ = 168 from more than 3 independent experiments. (g, right panel) The probabilities for finding either 0 (gray shaded area), 1 (green shaded area) or 2 (orange shaded area) labels as a function of Smc5/6 dimer over monomer ratio estimated on basis of the labeling efficiency of 68 ± 10%. The error bars indicate 95% confidence interval estimated using binomial proportion. Images and time traces in (a–d) are representative of five independent measurements. (e,f) are extracted from three independent experiments.

**Extended Data Fig. 6:**
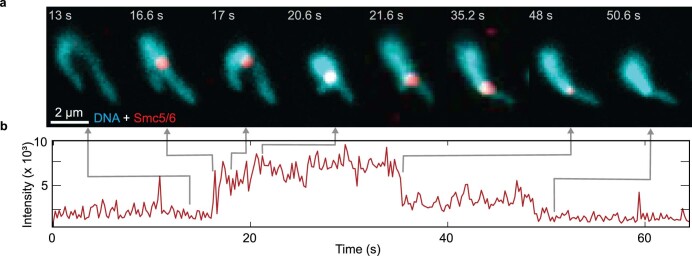
Two bleaching steps observed from labelled Smc5/6 at the stem of the DNA loop during loop extrusion. (a) Snapshots of DNA loop extrusion event showing labeled Smc5/6 at the stem of the extruded loop. (b) the corresponding time trace of Smc5/6 fluorescence intensity showing two-step bleaching. The arrows indicate the time points corresponding to the respective snapshots. (a,b) are the representative of five independent experiments.

We then labelled SNAP-tagged Smc5/6 complexes at the Nse4 subunit (Extended Data Fig. 1d; for gel source data, see Supplementary Fig. 2) with single Alexa 647 fluorophores (labelling efficiency 68 ± 10%; Methods) and co-imaged them with DNA during loop extrusion. This showed that the complex was positioned at the base of the extruded loop, further confirming an active extrusion process (Fig. 2a and Supplementary Video 3). To determine how many Smc5/6 complexes are required for extrusion, we monitored the fluorescence intensity of labelled loop-extruding complexes in real time (Fig. 2b–g). In the majority of cases (82%, *n*_loop_ = 168), the Smc5/6 signal atop DNA first increased in a single step, indicative of a Smc5/6–DNA binding event, followed by loop growth, and finally decreased in either a single or two consecutive steps (Fig. 2d,g, Supplementary Video 4 and Extended Data Fig. 5a–d) due to photobleaching (Extended Data Fig. 5e,f). A smaller fraction (18%) of loop-initiation events did not correlate with Smc5/6 signal, indicating looping by unlabelled complexes. Comparison of intensity distributions obtained from two- and one-step bleached events and background traces confirmed that the two-step bleaching process originates from no more than two fluorophore-labelled complexes (Fig. 2h). Interestingly, we observed a larger fraction (43%) of two-step bleaching events as compared with single-step (36%) (Fig. 2i). Because the labelling efficiency of Smc5/6 was below 100%, the correlation between the number of bleaching steps (one or two) and that of Smc5/6 complexes (monomer or dimer) is not linear. Importantly, a single bleaching step could arise either from a single labelled Smc5/6 or a Smc5/6 dimer with only one labelled complex. We therefore calculated the probability of observing zero (unlabelled), one and two bleaching steps for a labelling efficiency range of 68 ± 10% as a function of ‘dimer fraction’, where 0 indicates that all Smc5/6 complexes are monomers and 1 indicates that all are dimers (Extended Data Fig. 5g (right) and Methods). Interestingly, we found that the observed ratio most closely correlates with a 100% dimer fraction, indicating that loop-extrusion events are performed by Smc5/6 dimers. Furthermore, the photobleaching statistics obtained from the loop-extruding complexes labelled at the Nse2 subunit (Fig. 2j) with similar labelling efficiency (70 ± 10%) were also in good agreement with the expected ratios for dimers, indicating that the dimers are probably formed by two complexes rather than by a single complex carrying a duplicate of specific subunits. Real-time imaging of loop extrusion with labelled Smc5/6 under constant buffer flow (Extended Data Fig. 6) showed that these dimers were located at the stem of the loop during extrusion. We then questioned whether the dimeric state of Smc5/6 is necessary for loop extrusion or whether a single complex can extrude loops, but a second complex is frequently present due to the high likelihood of random complex–complex interaction. If so, the fraction of loop-extruding dimers is expected to decrease with decreasing protein concentrations. Interestingly, however, we observed that the fraction of two bleaching steps did not decrease even at tenfold lower protein concentration but instead remained consistently larger than the fraction of single bleaching steps (Fig. 2k). Furthermore, fitting the fraction of looped DNA observed at different Smc5/6 concentrations to a Langmuir–Hill equation showed that loop extrusion is stimulated by cooperative interactions, showing a Hill coefficient of *n*_H_ = 1.84, well above *n*_H_ = 1.0 (Fig. 2l and Methods). Taken together, these observations support the idea that the functional unit for Smc5/6 loop extrusion is a dimer of complexes. This contrasts with condensin, which extrudes loops as a single complex^10^, whereas cohesin has been suggested to extrude both as a monomer and dimer^11,12^.

**Fig. 3:**
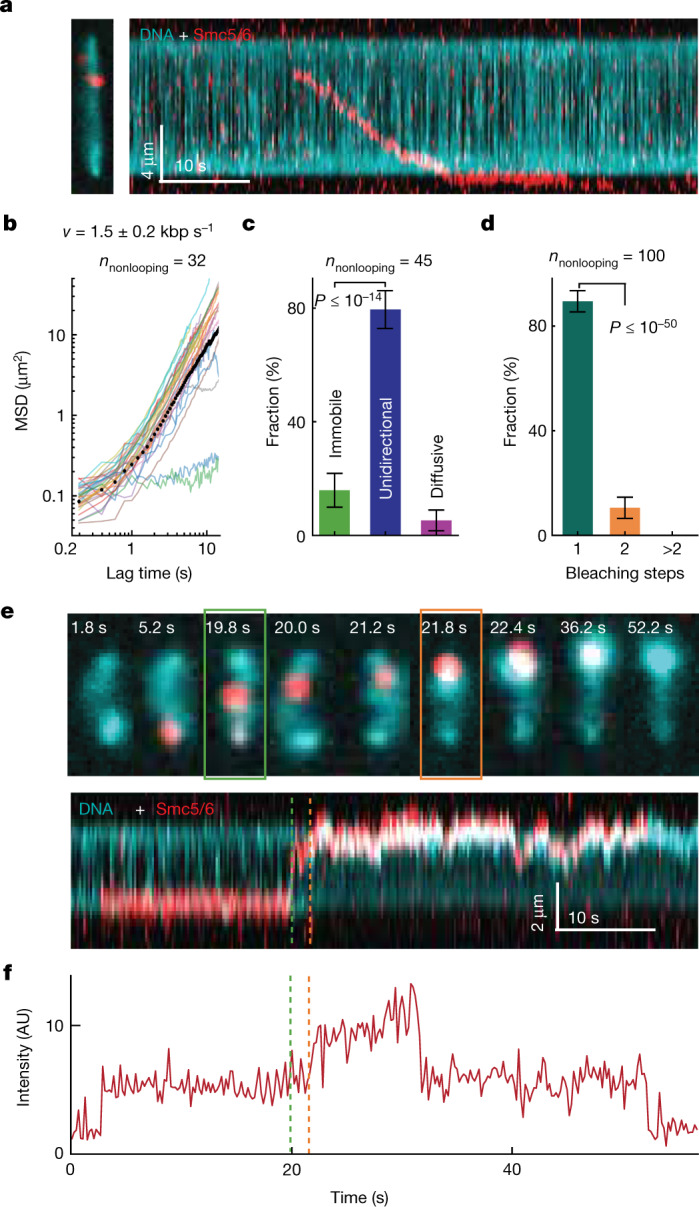
Single Smc5/6 complexes unidirectionally translocate along DNA. **a**, Overlaid snapshots (left) and kymograph (right) showing an example of a labelled Smc5/6 complex translocating on a DNA molecule. Representative of five independent experiments. **b**, MSD plots determined from kymograph trajectories of individual Smc5/6 nonlooping events (solid lines). **c**, Fractions of nonlooping Smc5/6 complexes that remained immobile or translocated directional or diffusive on DNA before dissociating from the DNA. **d**, Fractions of nonlooping Smc5/6 complexes that exhibited either one (green), two (orange) or more bleaching steps. Error bars in **c**,**d** indicate 95% confidence interval. Data in **b**—**d** were obtained from the respective *n*_nonlooping_ events over five independent measurements. **e**,**f** Snapshots (top) and kymographs (bottom) of Smc5/6 and DNA (**e**) and the corresponding time trace of Smc5/6 label intensity (**f**), showing an event where a translocating Smc5/6 started to extrude a DNA loop upon forming a dimer with another Smc5/6 from the solution. The dashed lines in **e**,**f** indicate the start of the Smc5/6 translocation (green) and the binding of the additional Smc5/6 (orange). Representative of three independent measurements.

**Extended Data Fig. 7:**
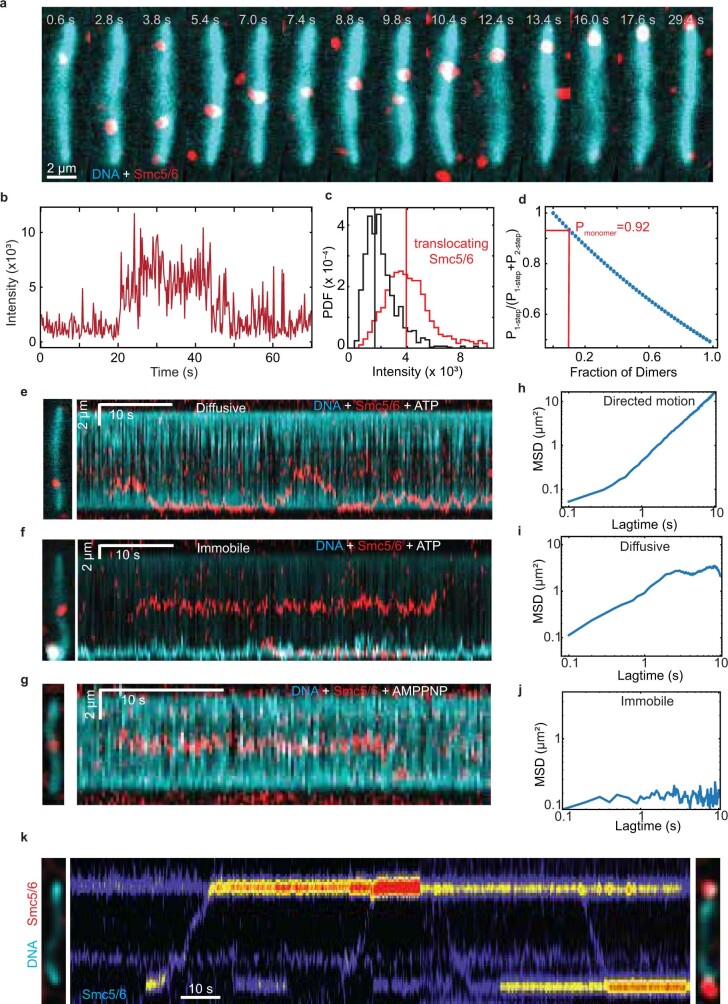
DNA Translocation by a single Smc5/6 complex. (a) Snapshots of labelled Smc5/6 (red) on DNA (cyan), showing a DNA translocation event, corresponding to Fig. 3a. (b) Intensity time trace of Smc5/6 depicted in (a) showing one-step bleaching. (c) Probability density functions (PDF) of fluorescence intensities for from Alexa647 signal on labeled Smc5/6 (red) and the background signal (black). (d) Calculated propability of observing single bleaching steps as a function of dimer fraction given that we experimentally only observe one-step and two-step bleaching events. Red line shows the experimentally observed value shown in Fig. 3d, indicating that 90% of events are likely come from single Smc5/6 complexes (P_monomer_) (e,f) Example kymographs of Smc5/6 (red) on DNA (cyan) in the presence of ATP showing diffusive and immobile characteristics. (g) Example kymograph of Smc5/6 remaining bound to DNA in the presence of AMP-PNP. (h,i,j) Mean square displacements (MSD) of Smc5/6 complexes displaying directed motion (h) corresponding to Fig. 3a, diffusive motion (i) extracted from the kymograph shown in (e), and immobile behaviour (j) corresponding to the kymograph in (f). (k) Example kymograph of Nse2-labeled Smc5/6 showing binding and translocation events leading to accumulation at the end of λ-DNA which is tethered on the surface. The snapshots of DNA (cyan) and Smc5/6 (red) before (left side of the kymograph) and after (right side of the kymograph) incubation of Smc5/6 for 1 h. Data in (a,b,c,e-g) are the representative of more than three independent experiments.

**Extended Data Fig. 8:**
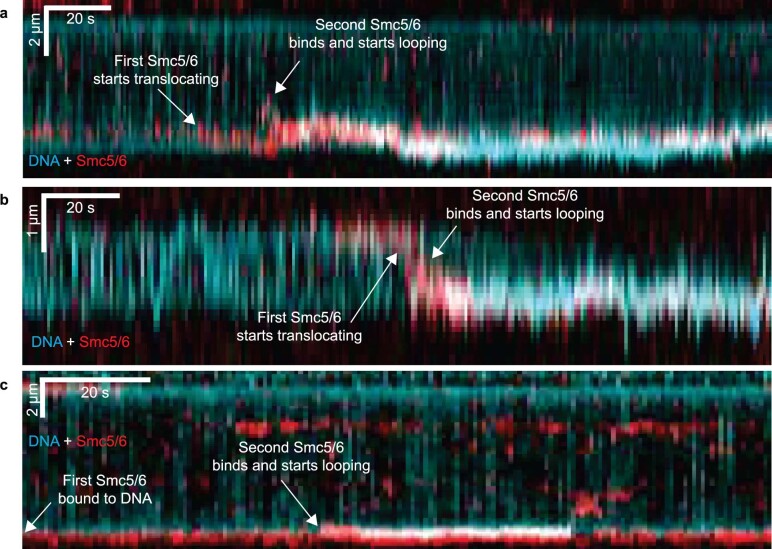
Dimerization of Smc5/6 on DNA results in initiation of loop extrusion. Example kymographs showing different types of events where loop extrusion (cyan) starts upon dimerization of Smc5/6 (red). (a) Two translocating Smc5/6 merge together and start loop extrusion. (b) A single Smc5/6 bound on the end of DNA starts translocating. A second Smc5/6 from the solution binds the first one and loop extrusion is initiated. (c) A single Smc5/6 is bound to one end of the DNA at the start of the measurement. Loop extrusion is initiated when a second Smc5/6 from the solution associates. Data in (a,b,c) are the representative of more than three independent experiments.

In addition to loop extrusion, we also observed that Smc5/6 can unidirectionally translocate along DNA (Fig. 3a and Supplementary Video 5) in an ATP-dependent manner (Extended Data Fig. 7g). Kymographs showed that around 80% (*n*_nonlooping_ = 45) of all nonlooping Smc5/6 translocated whereas a smaller fraction of molecules remained either stably bound at one position or randomly diffused along DNA (Fig. 3b,c and Extended Data Fig. 7a,e,f). Mean squared displacement (MSD) plots generated from tracking of labelled Smc5/6 in kymographs exhibit increasing slopes, consistent with directed motion of average translocation velocity (*v*) 1.5 ± 0.2 kbp s^–1^ (Fig. 3b and Extended Data Fig. 7h–j; *n*_tot_ = 32). We also found that translocation mostly stops when Smc5/6 reaches the DNA ends where it is stably bound over a long period of time, thus leading to the accumulation of proteins at these sites (Extended Data Fig. 7k). The photobleaching steps and fluorescence intensity distribution of translocating Smc5/6 (Fig. 3d and Extended Data Fig. 7b,c) show that roughly 92% of translocating units were labelled with a single fluorophore. By comparing this value with the calculated probability of single bleaching steps (Extended Data Fig. 7d), we found that our data match most closely with a fraction of monomers above 90%. Taken together, these findings indicate that a single Smc5/6 can translocate along DNA whereas a pair of complexes is required for DNA loop extrusion. In further support of this, 4% of all loop-extrusion events were initiated when a second Smc5/6 complex associated with a single translocating complex (Fig. 3e,f, Supplementary Video 6 and Extended Data Fig. 8).

**Fig. 4:**
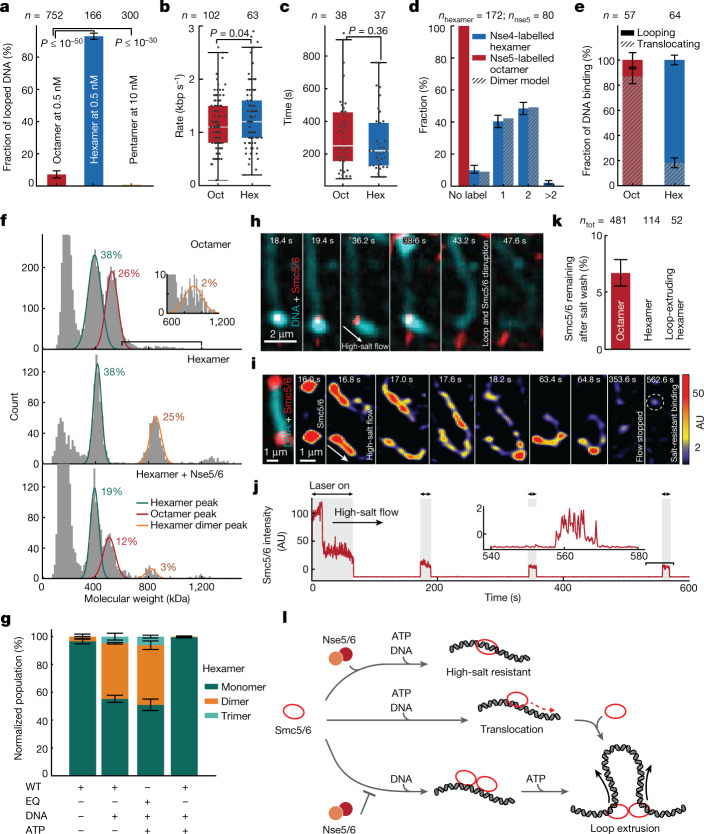
Nse5/6 downregulates loop extrusion by inhibiting dimerization of Smc5/6. **a**, Fractions of DNA molecules that formed loops following the addition of octameric (+Nse5/6), hexameric (–Nse5/6) or pentameric (–Nse2/5/6) complexes. **b**,**c**, Loop-extrusion rates (**b**) and loop dwell times (**c**) for octameric and hexameric complexes; median ± 1.5× IQR. **d**, Fraction of loop-extruding complexes with no label or one, two or more than two bleaching steps for Nse4-labelled hexamers and Nse5-labelled octamers. **e**, Fractions of translocating and looping events per DNA binding for octamers and hexamers. *P* ≤ 10^–28^, two-sided binomial test. **a**,**d**,**e**, Error bars indicate 95% confidence interval. Data in **a**—**e** are from indicated (*n*) events collected from more than three independent measurements. **f**, Histograms of mass distribution for octamer (top), hexamer (middle) and hexamer with additional Nse5/6 in a 1:1 ratio (bottom), measured in the presence of DNA without ATP. Peaks correspond to the molecular weights of hexamer (green), octamer (red) and dimer of hexamer (orange). Inset: zoomed-in peak centred at around 880 kDa. **g**, Fraction of monomers, dimers and trimers of WT and EQ mutant Smc5/6 observed under the indicated conditions, obtained from mass photometry; mean ± s.d. from three independent experiments. **h**, Snapshots of a loop (cyan) extruded by hexamers (red) on high-salt buffer flow showing an example of high-salt-induced loop disruption and subsequent protein dissociation. **i**, Snapshots of nonlooping Smc5/6 under high-salt buffer flow, showing that previously DNA-end accumulated complexes (16s) became redistributed along the DNA and subsequently dissociated (17s–353.6s), while a few molecules remained bound (562.6s). **j**, Time trace of fluorescence intensities from Nse2-labelled octamers during high-salt wash in **i**. Inset: zoomed-in trace towards the end of the high-salt wash. The laser was irradiated for short intervals (shaded area) to minimize photobleaching. Data in **h**–**j** representative of three independent experiments. **k**, Fractions of labelled octamers and hexamers remaining bound on DNA after high-salt wash; mean ± s.d. from three independent experiments. *n*_tot_, number of Smc5/6 before salt wash. **l**, Model of Smc5/6-mediated loop extrusion. Source data

**Extended Data Fig. 9:**
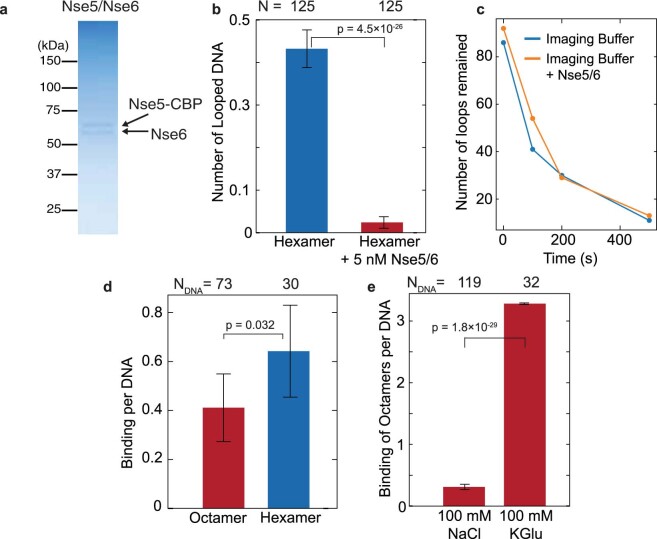
The effect of Nse5/6 on DNA loading, loop initiation and loop disruption. (a) CBB-stained SDS-PAGE for the Nse5/6 subcomplex. (b) Fraction of DNA that formed loops in the absence (blue) and the presence of purified Nse5/6 subcomplexes (5 nM, red) while the concentration of hexameric Smc5/6 complexes was held constant (0.5 nM). Note that Nse5/6 was mixed with hexamers shortly before introducing to the flow cell. (c) Number of loops remaining at different time points upon introducing buffer with or without a 10-fold excess of Nse5/6. t = 0 indicates the start of the buffer flow. The data shows that there are no additional loop disruption events induced by the presence of Nse5/6. (d) Number of DNA binding events of labeled octameric or hexameric Smc5/6 complexes per DNA molecule and per 1000 s. (e) Comparison between the numbers of DNA binding events obtained for octameric complexes in imaging buffers containing 100 mM NaCl or 100 mM Potassium Glutamate (KGlu). The error bars in b,d,e indicate 95% confidence interval estimated using binomial proportion.

Smc5/6 comprises three subunits (Nse2 and the Nse5/6 subcomplex) in addition to its pentameric core. To investigate the role of these subunits in Smc5/6-mediated loop extrusion, we purified the hexameric complex lacking Nse5/6 and the pentamer lacking Nse2 and Nse5/6 (Extended Data Fig. 1f,g,j) and determined their ATPase activities (Extended Data Fig. 1k). In subsequent single-molecule experiments with hexameric complexes we observed an increase of approximately 15-fold in looping probability in comparison with the WT octamer (Fig. 4a). In contrast to this difference in looping probability, the hexamer and octamer exhibited similar rates of loop extrusion (1.3 ± 0.6 and 1.1 ± 0.5 kbp s^–1^, respectively) and similar loop dwell times (270 ± 190 and 317 ± 229 s, respectively) (Fig. 4b,c). These findings suggest that Nse5/6 negatively regulates loop initiation but does not significantly influence extrusion dynamics after initiation. This is further supported by our finding that the addition of purified Nse5/6 to hexameric complexes before loop initiation reduced looping probability (Extended Data Fig. 9b), but the addition after loop initiation did not disrupt ongoing extrusion (Extended Data Fig. 9c). In the case of pentameric complexes we did not observe any looping events (*n*_tot_ = 300; Fig. 4a), even at protein concentrations tenfold higher than that used for analysis of the WT octamer, indicating that Nse2 is required for loop extrusion. This is a feature unique to Smc5/6-mediated loop extrusion, because other SMC complexes do not require an additional protein bound on the coiled-coil SMC arm for loop-extrusion activity.

To further clarify the regulatory role of Nse5/6 on loop initiation we collected photobleaching statistics of loop-extruding hexameric complexes labelled at the Nse4 subunit, and of Nse5-labelled octamers (Fig. 4d and Extended Data Figs. 1i and 9a). The observed ratios for Nse4-labelled hexamers were in good agreement with the expected ratios for Smc5/6 dimers, indicating that dimerization is required for loop extrusion also in the absence of Nse5/6. However, we did not observe any Smc5/6 fluorescence signal that correlates with loop-initiation events when analysing Nse5-labelled octamers (*n*_nse5_ = 80; Fig. 4d), indicating that loop-extruding Smc5/6 dimers lack Nse5/6. Furthermore, by analysis of the translocation and loop formation probability of Smc5/6 (Fig. 4e) per DNA loading (Extended Data Fig. 9d), we found that Nse5/6 increased the translocation probability of Smc5/6 from 18 ± 4% (for hexamer, *n*_tot_ = 64) to 87 ± 6% (for octamer, *n*_tot_ = 57). That said, Nse5/6 is not required for translocation because a significant fraction of Smc5/6 hexamers (18 ± 4%) can translocate along DNA. Knowing that translocation events are mostly performed by single complexes (Fig. 3d) whereas looping events require dimerization (Fig. 2h–l), and that loop-extruding Smc5/6 dimers do not contain Nse5/6 (Fig. 4d), we speculate that Nse5/6 inhibits loop initiation by inhibition of dimerization.

To test this hypothesis we used mass photometry and estimated the effect of Nse5/6 on Smc5/6 dimerization for hexameric and octameric complexes (Fig. 4f). We found that, in the absence of ATP and presence of DNA, Smc5/6 lacking Nse5/6 forms over tenfold more dimers (25% of total counts; Fig. 4f, middle) than Smc5/6 containing Nse5/6 (2%; Fig. 4f, top). Furthermore, the addition of purified Nse5/6 to hexameric complexes in a 1:1 ratio led to a reduction in dimerization (3%; Fig. 4f, bottom). The dimers detected in the presence of Nse5/6 (Fig. 4f, top, bottom) exhibited masses in the range expected for dimers of hexamers (MW_exp_ = 804 kDa) rather than that for dimers of octamers (MW_exp_ = 1,040 kDa), a finding consistent with our observation that loop-extruding Smc5/6 dimers lack Nse5/6 (Fig. 4d). Taken together, these data support the idea that Nse5/6 inhibits Smc5/6 dimerization.

**Extended Data Fig. 10:**
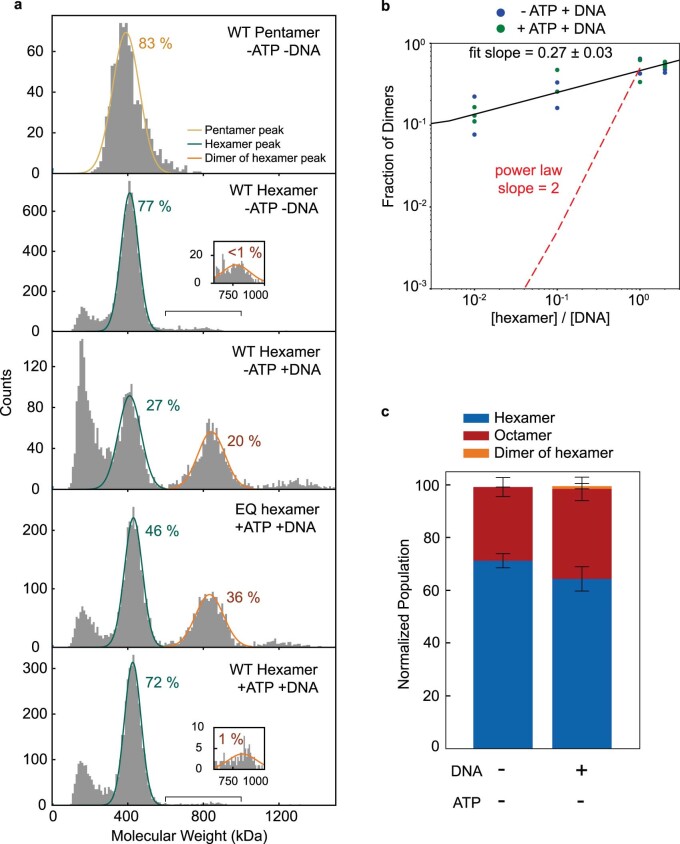
Characterization of Smc5/6 dimerization. (a) Mass distributions of pentameric (1^st^ in row) and hexameric Smc5/6 complexes in different conditions, including no ligand (2^nd^ in row), in the presence of DNA (3^rd^), ATP hydrolysis-deficient (EQ) mutant with ATP and DNA (4^th^), hexameric Smc5/6 with ATP and DNA (5^th^). The identified peaks exhibit mass distributions close to the molecular weight of pentamer (beige), single hexamer (green) and dimer of hexamer (orange). The inset panels show the zoom-in peak corresponding to the dimer of hexamers. The total number of counts for the fitted areas are displayed in percentage value compared to all detected events throughout the measurement period. (b) Fraction of dimers of hexamer EQ mutant in the absence (blue) or presence (green) of ATP with different protein:DNA ratios (2:1, 1:1, 1:10, and 1:100). The fraction of dimers is quantified as the total number of Smc5/6 that formed dimer normalized by the total number of Smc5/6. The red line with slope = 2 demonstrates that the expected outcome of a scenario where the dimer fraction observed reflects two monomeric Smc5/6 complexes binding to the same DNA without protein-protein interaction. Experimental data were fitted to a two-sided linear least-squares regression. Fitted slope of 0.27 signifies the theoretical power-law is not obeyed and dimer formed by Smc5/6 hexamer is due to additional interaction between the complexes, regardless of the presence of ATP. (c) Comparison of oligomeric states of octameric Smc5/6 complex in the absence (mean ± S.D. from 4 independent experiments) or presence of DNA (mean ± S.D. from 3 independent experiments) without ATP.

To gain further insight into the dimerization mechanism, we compared the dimer formation in the absence and presence of DNA, with or without ATP, and using the ATP hydrolysis-deficient EQ mutant (Fig. 4g). This showed that dimerization of hexameric complexes is significantly enhanced by the addition of DNA (over tenfold, 40 ± 5% of normalized population). This was also persistent when Smc5/6 was incubated with a large excess of DNA, supporting the idea that this enhancement reflects dimerization rather than two independent monomeric complexes binding to the same DNA (Extended Data Fig. 10b). Following the addition of ATP, however, the fraction of dimers was reduced back to a level similar to the DNA unbound state (2 ± 1%). For the ATP hydrolysis-deficient EQ mutant, the addition of ATP did not reduce the DNA-enhanced dimer fraction (43 ± 5% of normalized population). This is in line with the ATP-triggered loop extrusion and the subsequent dissociation from DNA. Taken together (Fig. 4a–g), these findings suggest that Nse5/6 negatively regulates loop initiation by inhibition of dimerization of hexamers, which is enhanced by DNA binding.

Our finding that Nse5/6 inhibits loop extrusion seems to be in conflict with the idea of Nse5/6 acting as a loader of Smc5/6 to chromatin^27,28^ and being required for high-salt-resistant topological loading of DNA^29,30^. To better understand the relation between loop extrusion and topological loading, and the role of Nse5/6 in these processes, we included a high-salt washing step in our loop-extrusion experiments. Specifically we first observed DNA binding, translocation (Extended Data Fig. 7k) and looping events by hexameric or octameric Smc5/6 and subsequently washed the flow cell with high-salt buffer (1 M NaCl) (Fig. 4h). This immediately disrupted all previously extruded loops (*n*_tot_ = 60) and, importantly, no loop-extruding complexes remained bound on DNA (*n*_tot_ = 52) (Fig. 4h,k). This suggests that the loop-extruding complexes were not topologically loaded on DNA. Interestingly, however, a minor fraction of nonlooping octameric complexes remained associated with DNA even after the high-salt wash after 1 h of incubation (7%, *n*_tot_ = 481; Fig. 4i–k and Supplementary Video 7), indicating that topological entrapment can occur but at a low frequency. In line with the requirement of Nse5/6 for high-salt-resistant topological loading^30^, no Smc5/6 remained after high-salt washing when hexamers lacking Nse5/6 were analysed (Fig. 4k). These findings show that, although Smc5/6 can extrude loops in the absence of Nse5/6 it cannot form high-salt-resistant binding to DNA without Nse5/6 which, in turn, indicates that loop extrusion occurs independently of topological entrapment of DNA.

In conclusion, our study shows that Smc5/6 is a DNA loop-extruding complex. Whereas the dynamics of Smc5/6-mediated loop extrusion exhibit similarities to cohesin and condensin, its mechanism involves distinct complex-specific features (Fig. 4l): Smc5/6 performs extrusion by cooperative pairs of complexes whereas single complexes translocate along DNA. Furthermore, Nse5/6 acts as a negative regulator of loop extrusion. Specifically, Nse5/6 inhibits loop extrusion at loop initiation by inhibition of dimerization of hexamers but has no influence on ongoing loop extrusion. Furthermore, Nse5/6 allows interaction of Smc5/6 with DNA in a high-salt-resistant manner, probably by topological entrapment of DNA, a binding mode not required for loop extrusion. Taken together, our findings indicate that Smc5/6 protects genome integrity using loop extrusion, opening up new avenues for dissection of the activities of this multifunctional complex.

## Methods

### Gene synthesis, subcloning and strain creation for Smc5/6 overexpression

Genes for all Smc5/6 subunits were synthesized by GeneArt Gene Synthesis (Thermo Fisher Scientific), with codon optimization and introduced into pJF2, pJF3, pJF4 and pJF5 yeast integrative vectors (kindly provided by J. Diffley) under the bidirectional *GAL1-10* promoter^29^. The TAP-tag sequence derived from pBS1479 (EUROSCARF) was introduced into the C terminus of Smc6 or Nse5 using standard methods. The following plasmids—CD373; *SMC5-GAL1-10-SMC6-TAP*, CD380; *NSE5-pGAL1-10-NSE6*, CD395; *NSE3-pGAL1-10-NSE4*, CD377; and *NSE1-pGAL1-10-NSE2—*were integrated into CB3245 using auxotrophic markers, then the *TOP1* gene was deleted using standard gene-replacement methods. For ATPase mutants, point mutations were introduced using standard methods at appropriate positions (Walker A (KE): Smc5K75E, Smc6K115E; Walker B (EQ): Smc5E1015Q, Smc6E1048Q)^31^. A codon-optimized SNAP tag was synthesized by GeneArt Gene Synthesis and introduced into pFA6a with a KAN marker. The SNAP tag was introduced into the C terminus of ectopic NSE2, NSE4 and NSE5 after the 6×His tag using standard methods. A list of yeast strains and plasmid DNA used in this study can be found in Supplementary Tables 1 and 2.

### Overexpression and purification of Smc5/6 and subcomplex Nse5/6

Overexpression strains were grown at 30 °C in 1 or 2 l of YEP-lactate medium to optical density (OD_600_) 0.8–1.0, then protein expression was induced for 4 h by the addition of 2% galactose. After harvesting, cells were disrupted using Freezer mill 6870 (SPEX), and proteins were extracted by the addition of one cell volume of IPP150 buffer (50 mM Tris-HCl pH 8.0, 150 mM NaCl, 10% glycerol, 0.1% IGEPAL CA-630, 1 mM DTT) containing 10 mM MgCl_2_ and complete EDTA-free protease inhibitor (Roche Applied Science), after which treatment with benzonase (Merck) was performed for 1 h at 4 °C. Cleared extracts were mixed with IgG Sepharose 6 FF (Merck) for 2 h at 4 °C and washed with IPP150 buffer. IPP150 buffer was then replaced with GF500 buffer (20 mM HEPES-NaOH pH 7.5, 500 mM NaCl, 10% glycerol, 0.1% IGEPAL CA-630, 1 mM DTT) and the resin treated with tobacco etch virus (TEV) protease (kind gift from H. Schüler) at 4 °C overnight. The fraction eluted by TEV proteinase treatment was diluted fourfold in CBB500 buffer (50 mM Tris-HCl pH 8.0, 500 mM NaCl, 1 mM Mg(CH_3_COO)_2_, 1 mM imidazole, 2 mM CaCl_2_, 1 mM DTT, 0.1% IGEPAL CA-630) supplemented with 1 M CaCl_2_ (30 µl for 40 ml of mixture) and incubated with calmodulin Sepharose 4B (Merck) for 2 h at 4 °C. After washing with CBB500 buffer, proteins were eluted using CEB500 buffer (50 mM Tris-HCl pH 8.0, 500 mM NaCl, 1 mM Mg(CH_3_COO)_2_, 1 mM imidazole, 20 mM EGTA, 1 mM DTT, 0.1% IGEPAL CA-630). The eluate was concentrated by around 50-fold using a Vivaspin20 ultrafiltration unit (100 K MWCO, Sartorius) concomitant with an exchange to STO500 buffer (50 mM Tris-HCl pH 8.0, 500 mM NaCl, 2 mM MgCl_2_, 0.5 mM tris(2-carboxyethyl)phosphine (TCEP), 10% glycerol, 0.1% IGEPAL CA-630). Concentration of the complex was determined by Bradford assay using bovine serum albumin (BSA) as standard. The integrity of purified Smc5/6 was tested using size-exclusion chromatography on a Superose 6 Increase 10/300 GL column (GE healthcare), pre-equilibrated with STO500 buffer and subsequent SDS–polyacrylamide gel electrophoresis (SDS–PAGE) analysis of eluted fractions (see Extended Data Fig. 1b for the WT octameric complex and Extended Data Fig. 1j for a hexameric complex lacking subunits Nse5 and Nse6).

### Fluorescent labelling of Smc5/6

The Smc5/6 complexes containing C-terminally tagged Nse2-6xHis-SNAP, Nse4-6xHis-SNAP or Nse5-6xHis-SNAP were overexpressed and purified using IgG Sepharose 6 FF as described above. After TEV protease cleavage, the eluate was concentrated by around 50-fold using a Vivaspin20 ultrafiltration unit (100 K MWCO, Satorius) concomitant with an exchange to STO500 buffer. For fluorescent labelling, the eluate was mixed with 20 µM SNAP-Surface Alexa Flour 647 (NEB) in 50 µl of STO500 buffer supplemented with 50 mM DTT and incubated overnight at 4 °C. The mixture was concentrated by approximately tenfold using an Amicon Ultra centrifugal filter (100 K MWCO, Merck) concomitant with buffer exchange to fresh STO500 for removal of free Alexa Fluor 647.

### Labelling efficiency estimation

Labelling efficiency was calculated in two steps using Smc5/6 containing Nse4- (or Nse2-) 6xHis-SNAP-Alexa 647. The amount of Smc5/6 was first estimated by Bradford assay using BSA as standard, which was 7.56 ± 0.5 µM. The amount of label (Alexa 647) was then estimated by comparison of both absorption and fluorescence intensity of a known concentration (for example, 1 µM) of Alexa 647 in the same storage buffer used for labelled Smc5/6. Both absorption and fluorescence measurements yielded a labelling efficiency of 68 ± 10%, within their respective error.

### ATPase assay

Smc5/6 (0.5 µl, final concentration 30 nM) was incubated with 4 nCi [α-^32^P]ATP in 5 µl of the reaction buffer (50 mM Tris-HCl pH 7.6, 40 mM KCl, 1 mM MgCl_2_, 1 mM DTT, 0.1 mg ml^–1^ BSA) containing 1 mM ATP and various concentrations of pRS316 at 30 °C. Aliquots (1 µl) were collected every 30 min for 90 min and mixed with 1.5 µl of 1% SDS to stop the reaction. Then, 1 µl of the mixture was spotted on TLC PEI cellulose F plates (MERCK) and developed in 1 M HCOOH/0.5 M LiCl. Radiolabelled ATP and ADP were quantified using a LAS-3000 imager (Fujifilm). ATPase rates at each DNA concentration were calculated by linear regression using the least-squares method. Maximum ATPase rate and 95% confidence interval for the WT complex were obtained by fitting of a stimulatory dose–response model to experimental data by nonlinear regression using Prism 9 software (GraphPad).

### Highly inclined optical light sheet microscopy and data collection

A custom-built microscope was used for single-molecule visualization of DNA and labelled Smc5/6. Lasers with wavelengths of 638 nm (Cobolt) and 561 nm (Coherent) were coupled to a Zeiss (AxioVert200) microscope body through a single-mode fibre in wide-field illumination mode with the potential of changing the illumination angle. This setup allowed us to use highly inclined optical light sheet illumination using a total internal reflection fluorescent objective (alpha-Plan-APOCHROMAT ×100/1.46 numerical aperture, oil) for selective imaging of DNA and Smc5/6 while minimizing out-of-focus fluorescence background and bleaching. The fluorescence signal from the sample was spectrally selected by a dichroic filter (no. t405/488/561/640rpc2, Chroma) and recorded with a sCMOS (PCO edge 4.2) camera. Light from the excitation lasers (638 and 561 nm) was additionally suppressed using a multiband notch filter (no. NF03-405/488/561/635E-25, Semrock) located in front of the camera. For simultaneous imaging of DNA and Smc5/6, alternative excitation between the 561 and 638 nm lasers was used through electronic triggering of an acoustic-optic tunable filter (MPDSnCxx-ed1-18 and AOTFnC_MDS driver from AA-Optoelectronic). The temperature of the flow cell was controlled by adjustment of electric current sent through a self-adhesive heating foil (Thermo TECH Polyester Heating foil self-adhesive 12 V DC, 12 V AC 17 W IP rating IPX4 (L × W) 65 × 10 mm^2^) attached to the top of the glass slide. The temperature was set to 30 °C for all experiments unless stated otherwise. A custom-written python software was used for recording, storing and visualization of data. Specifically, we utilized PyQtGraph (https://github.com/pyqtgraph/pyqtgraph) and napari (https://github.com/Napari/napari)^32^ for visualization and export of images. Typically, images were recorded at 100 ms exposure time per frame for a duration of 1,000–2,000 s unless stated otherwise.

### Mass photometry experiments

Mass photometry measurements were carried out on a TwoMP device (Refeyn). Glass coverslips were rinsed in the following order: deionized water, 50% isopropanol, deionized water, 50% isopropanol and water, followed by drying in a clean nitrogen stream. The flow chamber was assembled as described in ref. ^33^. Before measurements, samples were diluted to a final concentration of 10 nM and incubated in assay buffer containing 40 mM Tris-HCl pH 7.5, 100 mM potassium glutamate and 7.5 mM MgCl_2_ at 30 °C for 10 min. All buffers used for mass photometry experiments were filtered with a 0.22 µm syringe filter (with a polyvinylidene difluoride membrane, Merck Millex). Mass photometry experiments with oligonucleotides were performed with 10 nM Smc5/6 hexameric or octameric complexes (WT or EQ mutant) and 5 nM 200 base pair (bp), linear double-stranded DNA(5′-TGGTTTTTATATGTTTTGTTATGTATTGTTTATTTTCCCTTTAATTTTAGGATATGAAAACAAGAATTTATCTGGTTTTTATATGTTTTGTTATGTATTGTTTATTTTCCCTTTAATTTTAGGATATGAAAACAAGAATTTATCTGGTTTTTATATGTTTTGTTATGTATTGTTTATTTTCCCTTTAATTTTAGGATATG-3′), unless stated otherwise. If stated, 10 nM Nse5/6 and 2.5 mM ATP were supplemented to the reaction. Immediately after injection of the sample into the flow chamber, images were acquired for 60 s at 135 Hz in all measurements. After each measurement the chamber was rinsed in the following order: water, 1 M NaCl, water and assay buffer. Data analysis was performed by DiscoveryMP (Refeyn). For contrast to mass conversion, the mass of the Smc5/6 hexamer EQ mutant without DNA and ATP was used as calibrant on the same day as each measurement. All samples were measured at least three times, unless stated otherwise.

### Single-molecule loop-extrusion assay

#### Flow cell preparation

The single-molecule assay used throughout this work was prepared as described previously^9,25,30^ with the following slight modifications: microscope slides were cleaned with acid piranha (sulfuric acid (five parts) and hydrogen peroxide (one part)) and silanized with 3-[(2-aminoethyl)aminopropyl] trimethoxysilane in methanol containing 5% glacial acetic acid, which leaves free amine groups on the surface. Slides were then treated with 5 mg ml^–1^ methoxy-PEG-N-hydroxysuccinimide (no. MW 3500, Laysan Bio) and 0.05 mg ml^–1^ biotin-PEG-N-hydroxysuccinimide (no. MW3400, Laysan Bio) in 50 mM borate buffer pH 9.0. The pegylation step was repeated five times to minimize nonspecific surface sticking of proteins. Pegylated slides were dried under a gentle flow of nitrogen, sealed and stored at −20 °C until further use. Flow cells were then assembled with the functionalized glass slides as previously described^9^. Each flow cell contained one inlet and two outlet channels to facilitate buffer flow application perpendicular to the axis of the immobilized DNA. The fluidic channels were first incubated with 100 nM streptavidin in T50 buffer (40 mM Tris-HCl pH 7.5, 50 mM NaCl) for 1 min and then washed thoroughly with T50 buffer. Subsequently, 10 pM Phage λ-DNA molecules, labelled with biotins at both ends^31^, were introduced to the flow cell at a constant speed of 3 μl min^–1^, resulting in surface immobilization of DNA molecules with relative DNA extensions ranging from 0.1 to 0.6. Unbound DNA molecules were later washed out. To minimize unwanted surface sticking of Smc5/6, the flow cell was further passivated by incubation with 0.5 mg ml^–1^ BSA for 5 min.

#### Single-molecule imaging of Smc5/6-mediated loop extrusion

Real-time imaging of Smc5/6-mediated loop extrusion was carried out as follows. The imaging buffer (40 mM Tris-HCl pH 7.5, 100 mM NaCl, 7.5 mM MgCl_2_, 0.5 mg ml^–1^ BSA, 1 mM TCEP, 2 mM ATP, 200 nM SxO, 30 mM d-glucose, 2 mM trolox, 10 nM catalase, 37.5 µM glucose oxidase) containing Smc5/6 (2 nM, unless stated otherwise) was introduced into the flow cell at a flow rate of 30 µl min^–1^ for 1 min and flow was stopped thereafter. For the side-flow experiment, a larger volume (200–300 µl) of the sample of the same composition was continuously flowed into the channel at 15 µl min^–1^. If only SxO-stained DNA was imaged, only the 561 nm laser was used, at an intensity of 0.1 W cm^–2^ whereas, for dual-colour imaging, SxO-stained DNA and Alexa 647-labelled Smc5/6 were imaged by alternating excitation using 531 nm (0.1 W cm^–2^) and 638 nm (about 150 W cm^–2^) lasers.

#### Single-molecule analysis of high-salt-resistant Smc5/6–DNA binding

For the estimation of high-salt-resistant DNA entrapment by octameric Smc5/6 containing Nse5/6, Nse2-SNAP-Alexa 647-labelled Smc5/6 was incubated with DNA in our single-molecule assay for 1 h at 2 nM concentration, which led to the accumulation of Smc5/6 at the ends of DNA (Extended Data Fig. 7k). We further enhanced the number of Smc5/6 bindings on DNA using 100 mM potassium glutamate rather than 100 mM NaCl in the imaging buffer (Extended Data Fig. 9e). Subsequently we exchanged the imaging buffer with 1 M NaCl containing 500 nM SxO at a flow rate of 10 µl min^–1^ and imaged the labelled Smc5/6 during high-salt washing to estimate the number of remaining Smc5/6 molecules after washing. To minimize the effect of bleaching, and thus to avoid underestimation of the number of Smc5/6, labelled Smc5/6 was imaged for short intervals during high-salt washing. In the case of hexameric complexes the same experimental conditions were used, including 2 nM protein concentrations and Nse4-SNAP-Alexa 647-labelled hexamers.

### Data analysis

Fluorescence images were analysed using a custom-written python software^32,34^ Regions containing λ-DNA molecules were chosen manually, cropped and saved into TIFF format. For snapshots of the molecules shown in this paper (Fig. 1e and others), the background was subtracted using the 'white_tophat' filter in scipy^33^. For further quantification of fluorescence intensity, an additional median filter (radius two pixels) was applied. Snapshots of labelled Smc5/6 (Fig. 2a,b,e) were denoised using a machine learning-based method (Noise2void) for better visualization^34^. For further quantification of fluorescence intensity (that is, for building of kymographs), however, the same median filter used for the DNA was employed. From the median filtered images, kymographs were subsequently built by summation of fluorescence intensity greater than 11 pixels along lines centred around the DNA axis (Fig. 1g,i). Intensities in the kymograph were normalized such that values outside of DNA approached zero. Each vertical pixillated line in the kymograph corresponds to one time point (one image-frame) of the image sequence.

#### Estimation of DNA size

The position of the DNA punctum—the centre position of a DNA loop—in the DNA kymograph was found using the ‘find_peaks’ algorithm in scipy, which determines the positions of local peak maxima for each line of the kymograph. We selected the most intense peak along each line on the kymograph. The intensity of DNA puncta—the entire region containing the DNA loop—was obtained by summing over the area of a square with side length seven pixels and centred around the punctum position, termed Int_loop_. The remaining DNA regions outside of the puncta were separated into two categories, termed Int_up_ and Int_down_, where ‘up/down’ is the intensity from the DNA region above/below the puncta (Fig. 1h). The amounts of DNA in, above and below the loop were estimated by multiplying the fraction of the intensity in the respective regions by 48.5 kbp (the length of the used lambda DNA in bp):

$${\rm{DNA}}\,{\rm{size}}\,{\rm{in}}\,{\rm{the}}\,{\rm{loop}}\,({\rm{bp}}),{I}_{{\rm{loop}}}=\frac{{{\rm{Int}}}_{{\rm{loop}}}\times 48502}{{\rm{Total}}\,{\rm{DNA}}\,{\rm{intensity}}},$$

$${\rm{DNA}}\,{\rm{size}}\,{\rm{above}}\,{\rm{the}}\,{\rm{loop}}\,({\rm{bp}}),{I}_{{\rm{up}}}=\frac{{{\rm{Int}}}_{{\rm{up}}}\times 48502}{{\rm{Total}}\,{\rm{DNA}}\,{\rm{intensity}}},$$

$${\rm{DNA}}\;{\rm{size}}\;{\rm{below}}\;{\rm{the}}\;{\rm{loop}}\,({\rm{bp}}),{I}_{{\rm{down}}}=\frac{{{\rm{Int}}}_{{\rm{down}}}\times 48502}{{\rm{Total}}\,{\rm{DNA}}\,{\rm{intensity}}}$$

Changes in DNA size in the respective regions were plotted as a function of time, as shown in Fig. 1h,j. These data were plotted, together with the smoothed data, using a Savitzky–Golay second-order filter and window size of 50 points (solid curve, Fig. 1h,j). Looking at Fig. 1n, the loop extrusion was termed two-sided if the increase in *I*_loop_ was correlated with a decrease in both *I*_up_ and *I*_down_, otherwise the events were deemed one-sided. To estimate the rate (*k*) of loop extrusion, the initial 5 s of the loop growth curve was fitted (Fig. 1l and Extended Data Fig. 3b) with a line *I*_loop_ = *kt* + *c*, where *c* compensates for the initial DNA amount before loop extrusion. Time traces of loop-extrusion rates were then obtained using $k\left(t\right)=\left[{I}_{{\rm{loop}}}\left(t+dt\right)-\,{I}_{{\rm{loop}}}\left(t-dt\right)\right]/2dt$, with *dt* = 1 s and consequent smoothing with the Savitzky–Golay filter as described above (Extended Data Fig. 3c). For force estimation, relative extension was first calculated as ${R}_{{\rm{ext}}}=48502d/\left(\left({I}_{{\rm{up}}}+{I}_{{\rm{down}}}\right){L}_{{\rm{C}}}\right)$, where *L*_C_ = 16 μm is the contour length of λ-DNA and *d* is the end-to-end distance of double-tethered DNA (in μm) (Extended Data Fig. 3d,e). Subsequently, relative extension was converted to force (Extended Data Fig. 3f) via linear interpolation of the force–extension curve obtained by magnetic tweezer force spectroscopy^35^. The force at maximum loop size was taken as the stalling force (Fig. 1m).

#### Estimation of number of Smc5/6

For determination of the number of Smc5/6 required for loop extrusion (Fig. 2c,d,f,g), image sequences recorded using ALEX were used to build kymographs of DNA and labelled Smc5/6 intensities. The areas used to determine intensities of DNA puncta were then utilized to determine Smc5/6 intensities and build intensity time traces, which were then used to count bleaching steps (Fig. 2d) and to build intensity histograms (Fig. 2h). For determination of the photobleaching statistics shown in Fig. 2i we included only molecules for which we observed loop initiation during the recording interval—that is, loops already initiated before recording started were excluded from analysis. Bleaching times for one-step bleaching (Δ*τ*_11_) and those for two-step bleaching (Δ*τ*_21_, Δ*τ*_22_) were than used to calculate the respective average bleaching times and to build the histogram shown in Extended Data Fig. 5a,c,e,f.

To quantify the fluorescence intensity of labelled Smc5/6, those Smc5/6 complexes that did not perform loop extrusion were localized and separately categorized as ‘nonlooping Smc5/6’. The intensity trace of Smc5/6 was calculated by summing intensities over the square centred around the localized position of Smc5/6 in the kymograph. Smc5/6 molecules that bound and were stuck at the end of the DNA near the PEG surface were not considered for further analysis.

For estimation of the number of Smc5/6 during the high-salt wash, the intensity of a single label was estimated from those surviving at the end of the salt wash (Fig. 4j, inset). The number of Smc5/6 that survived the high-salt wash was further verified from the number of bleaching steps with the measurement at 100 mM NaCl post high-salt wash.

The MSD of nonlooping Smc5/6 molecules was calculated from traces of their respective positions determined using trackpy^36^. Positions were tracked until individual Smc5/6 had reached either end of a DNA construct. The MSD (Fig. 3b) was fitted with a directed motion equation: ${\rm{MSD}}(t)={v}^{2}{t}^{2}+4Dt$, where *v* is mean velocity, *D* is the diffusion coefficient and *t* is lag time. The velocity (in μm s^–1^) obtained from these fits was then converted to kbp s^–1^ as $v\left(\frac{kbp}{s}\right)=v\left(\frac{\mu {\rm{m}}}{{\rm{s}}}\right)\times 48.5\,{\rm{k}}{\rm{b}}{\rm{p}}/{L}_{{\rm{a}}{\rm{v}}{\rm{g}}}$, where *L*_avg_ = 9 μm is the average end-to-end distance of those DNAs on which translocation was observed.

### Langmuir–Hill plot

Loop-extrusion experiments were performed at different concentrations of WT Smc5/6. These measurements, each of 10 s duration, were recorded after an incubation period of 15 min. At this time point an equilibrium state is reached and the fraction of looped DNA remains almost constant. The fraction of DNA constructs that had formed loops ($f[L]$) is determined and plotted as a function of Smc5/6 concentration ($[L]$) and fitted with the Hill–Langmuir function: $f([L])={\left[L\right]}^{n}/\left({\left({K}_{{\rm{a}}}\right)}^{n}+{\left[L\right]}^{n}\right)$, where *K*_a_ is the concentration of Smc5/6 at which half of the DNA is looped and *n* is the Hill coefficient. DNA molecules of end-to-end distance greater than 10 µm were not counted for this analysis, because they are unlikely to act as DNA substrates for loop extrusion due to the high tension/stall force on the stretched DNA (Fig. 1m and Extended Data Fig. 4b).

### Probability of bleaching steps derived from dimer:monomer ratio

We determined the probability *P*(*n*) of observing either *n* = 0, 1 or 2 bleaching steps as a function of dimer fraction *x* with the following formula:

$$P(2)={p}^{2}x,P(1)=2(1-p)px+px,P(0)=(1-p)(1-x)+{(1-p)}^{2}x,$$

where *p* represents labelling efficiency. The respective errors were calculated using $\sigma \left(P\left(n\right)\right)=\pm \frac{\text{d}P\left(n\right)}{\text{d}p}{\sigma }_{p}$, where *σ*_*p*_ is the error of labelling efficiency.

### Quantification and statisitcal analysis

The fitting of curves in Figs. 2l and 3b and Extended Data Figs. 3b and 10b was done with the Scipy package in python (v.3.9)^35^. Smoothing of data in Fig. 1h,j and Extended Data Fig. 3a,c–f was done with interpolation by the Savitzky–Golay method in Scipy using 50 data points. Error bars with 95% confidence interval in Figs. 1k, 2i–k, 3c,d and 4a,d,e and Extended Data Fig. 9b,d,e were calculated using the ‘binomial proportion confidence interval’. Box whisker plots in Figs. 1l,m and 4b,c contain the respective median values (horizontal white lines), with the box extending from Q1–Q3 quartile values of the data and bars extending no more than 1.5× IQR from the edges of the box. *P* values throughout the manuscript were calculated with a two-sided Student’s *t*-test unless otherwise stated.

### Reporting summary

Further information on research design is available in the Nature Portfolio Reporting Summary linked to this article.

## Online content

Any methods, additional references, Nature Portfolio reporting summaries, source data, extended data, supplementary information, acknowledgements, peer review information; details of author contributions and competing interests; and statements of data and code availability are available at 10.1038/s41586-023-05963-3.

## Supplementary information

Supplementary Figures Supplementary Fig. 1 contains uncropped SDS–PAGE gel data from Extended Data Fig. 1a–c,j. Supplementary Fig. 2 contains uncropped CBB-stained SDS–PAGE gel data from Extended Data Figs. 1d–i and 9a.

Reporting Summary

Supplementary Tables Supplementary Tables 1 and 2 contain lists of the yeast strains and plasmid DNA used in the study.

Supplementary Video 1 Real-time imaging of DNA loop extrusion by Smc5/6 under sideways flow application. The video corresponds to Fig. 1e.

Supplementary Video 2 DNA loop extrusion by Smc5/6 in the absence of buffer flow. The representative video and corresponding kymograph of a loop-extrusion event are shown. The video corresponds to Fig. 1f–h.

Supplementary Video 3 Side-flow visualization of DNA (cyan) loop extrusion by single fluorophore-labelled Smc5/6. The Smc5/6 (red) binds on the DNA at around 7 s and starts loop extrusion. The Smc5/6 remains at the stem of the loop before finally bleaching at around 151 s. The video relates to Fig. 2a.

Supplementary Video 4 DNA loop extrusion by labelled Smc5/6 in the absence of buffer flow. Kymograph and image sequences from DNA (top), labelled Smc5/6 (middle) and their merge (bottom). The video corresponds to Fig. 2e–g.

Supplementary Video 5 Unidirectional translocation of a single Smc5/6 on DNA. The time-lapse video of DNA and Smc5/6 (right) and corresponding kymograph (left) are shown. The video corresponds to Fig. 3a.

Supplementary Video 6 A single translocating Smc5/6 complex dimerizes with another Smc5/6 whereupon loop extrusion is initiated. The video corresponds to Fig. 3e,f.

Supplementary Video 7 Nse5/6 enables high-salt-resistant DNA binding of Smc5/6. The video shows labelled Smc5/6 accumulated at the ends of DNA after incubation for 1 h followed by a high-salt wash. Following the introduction of high-salt buffer, Smc5/6 start to diffuse away from the DNA ends and becomes redistributed along the DNA. Gradual dissociation of Smc5/6 from the DNA was observed thereafter, with a small fraction of the protein remaining bound after 15 min of incubation in the high-salt buffer. The video corresponds to Fig. 4i,j.

## Data Availability

Micrographs and microscopy images for selected molecules used in the figures can be found at 10.5281/zenodo.7636744. Statistical data can be found at 10.5281/zenodo.7636758. Source data are provided with this paper. Any other original imaging data reported in the paper are available on request.

## Source data

Source Data Fig. 1
Source Data Fig. 4
Source Data Extended Data Fig. 1

## Code availability

The Python-based data analysis source code used for analysis of data is available at https://github.com/biswajitSM/ResearchDataSmc56Looping. Kymograph analysis and processing of images were done with https://github.com/biswajitSM/LEADS.
