# Supplementary material for: The Smc5/6 complex is a DNA loop-extruding motor
Source: Nature. 2023 Apr 19;616(7958):843–8. doi: 10.1038/s41586-023-05963-3 (PMC10132971; doi:10.1038/s41586-023-05963-3)

nature portfolio

https://doi.org/10.1038/s41586-023-05963-3

Supplementary information

The Smc5/6 complex is a DNA loop-extruding motor

In the format provided by the authors and unedited

a. Extended Data Figure 1a

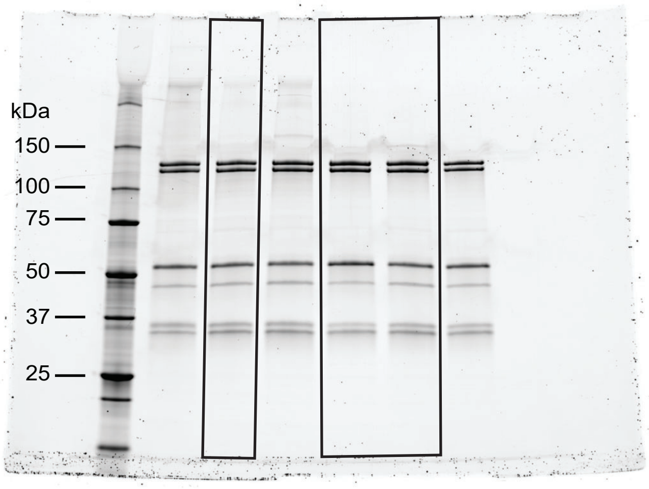

b. Extended Data Figure 1b

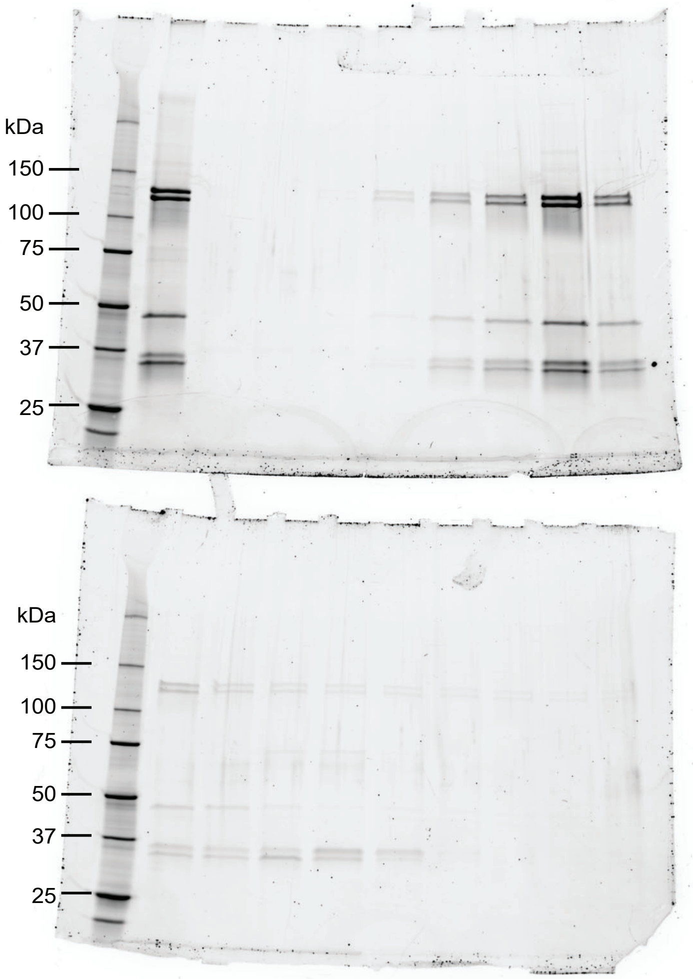

c. Extended Data Figure 1j

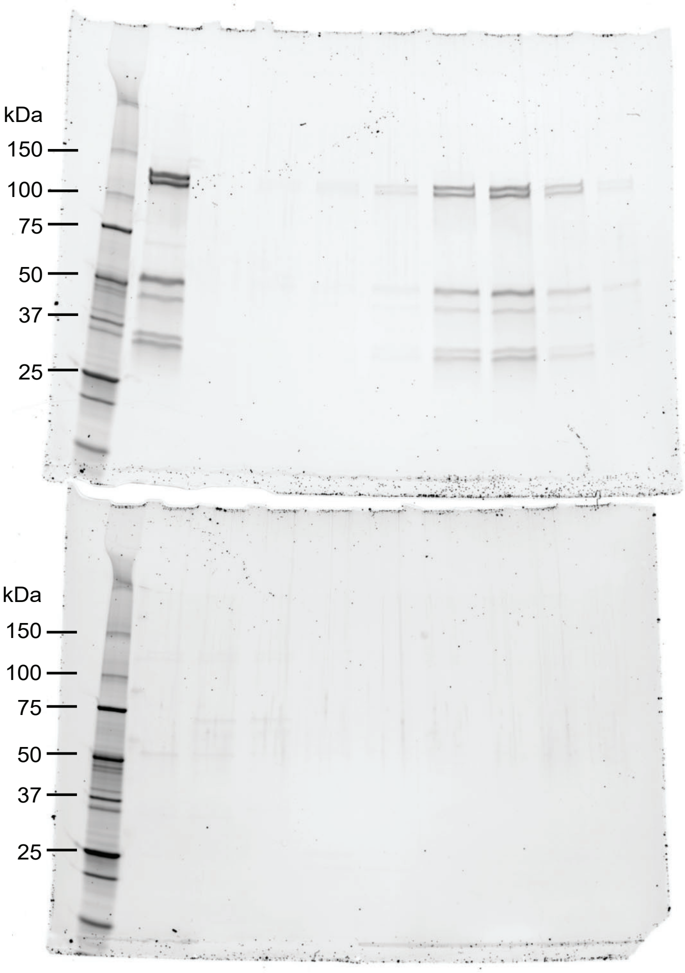

a. **Extended Data Figure 1d**

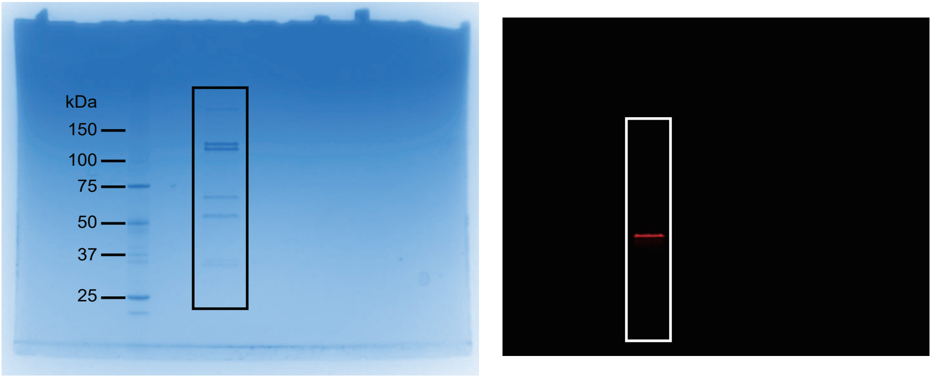

b. **Extended Data Figure 1e**

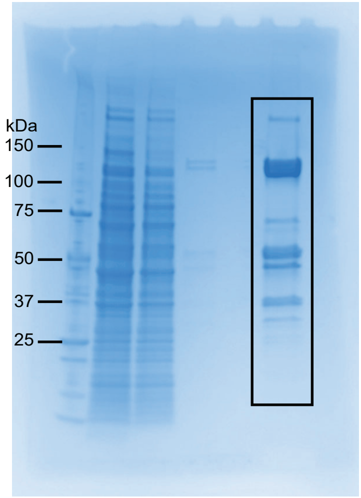

c. **Extended Data Figure 1f**

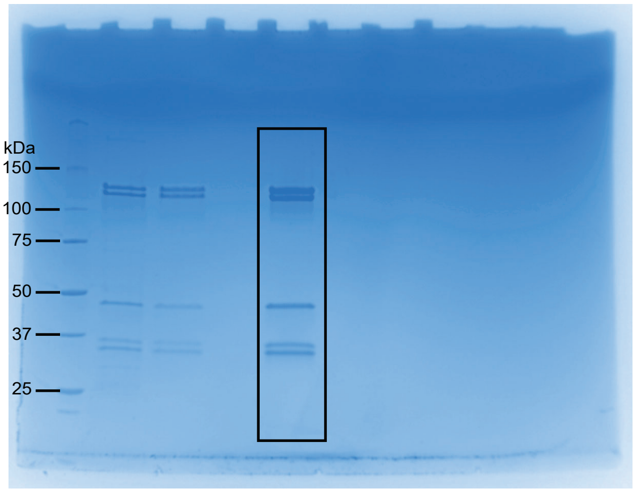

d. **Extended Data Figure 1g**

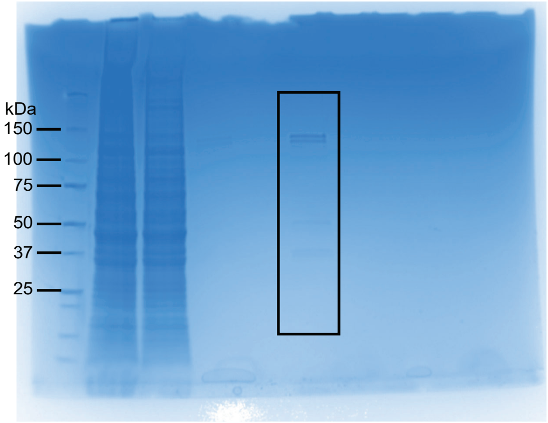

e. **Extended Data Figure 1h**

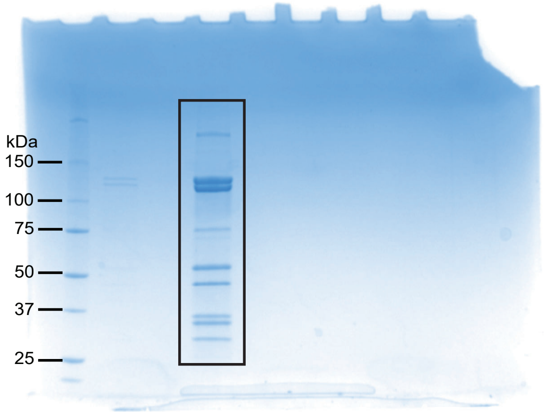

f. **Extended Data Figure 1i**

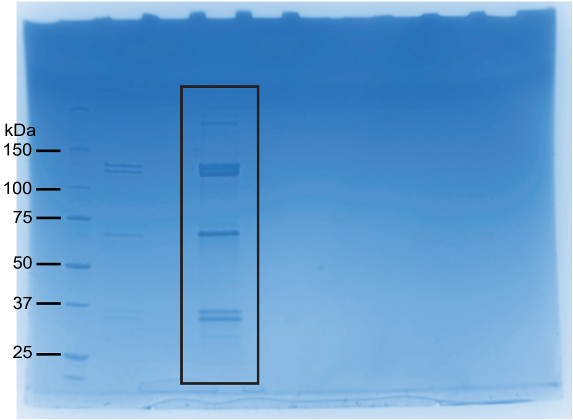

g. **Extended Data Figure 9a (Nse5-CBP/Nse6)**

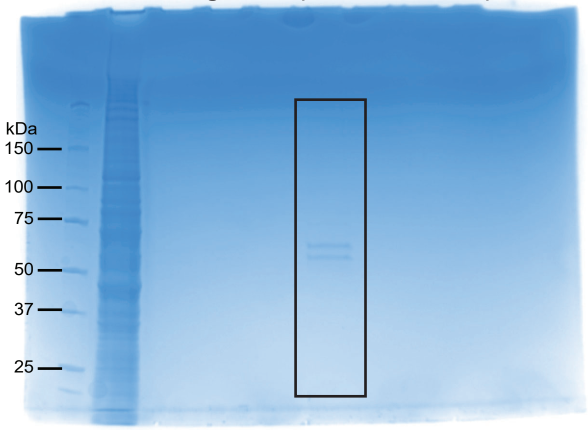

Supplement: Supplementary file 1 — Supplementary Fig. 1 contains uncropped SDS–PAGE gel data from Extended Data Fig. 1a–c,j. Supplementary Fig. 2 contains uncropped CBB-stained SDS–PAGE gel data from Extended Data Figs. 1d–i and 9a. [file 41586_2023_5963_MOESM1_ESM.pdf]
