# Supplementary material for: The Smc5/6 complex is a DNA loop-extruding motor
Source: Nature. 2023 Apr 19;616(7958):843–8. doi: 10.1038/s41586-023-05963-3 (PMC10132971; doi:10.1038/s41586-023-05963-3)
Supplement: Supplementary file 3 — Supplementary Tables 1 and 2 contain lists of the yeast strains and plasmid DNA used in the study. [file 41586_2023_5963_MOESM3_ESM.docx]

Supplementary Information for

***The Smc5/6 complex is a DNA loop extruding motor***

Biswajit Pradhan^1^†, Takaharu Kanno^2,3^†, Miki Umeda Igarashi^2,3^, Mun Siong Loke^1^, Martin Dieter Baaske^1^, Jan Siu Kei Wong^1^, Kristian Jeppsson^2,3^, Camilla Björkegren^2,3^*, Eugene Kim^1^*.

Corresponding authors. Email: [Camilla.Bjorkegren@ki.se](mailto:Camilla.Bjorkegren@ki.se), [Eugene.Kim@biophys.mpg.de](mailto:Eugene.Kim@biophys.mpg.de)

**Table S1. Yeast strains used in this study.**

All strains are of W303 origin, *ade2-1 trp1-1 can1-100 leu2-3, 112 his3-11, 15 ura3-1 and RAD5*, with additional modifications listed below.

| *Strain* | *Modification* |
| --- | --- |
| CB3245 | *MATa pep4∆::HPH* |
| CB3573 | *MATa pep4∆::HPH top1∆::NAT*  *leu2::LEU2pRS305-NSE1-pGAL1-10-NSE2*  *his3::HIS3pRS303-NSE3-pGAL1-10-NSE4*  *ura3::URA3pRS306-SMC5-pGAL1-10-SMC6-TAP* |
| CB3580 | *MATa pep4∆::HPH top1∆::NAT*  *trp1::TRP1pRS304-NSE5-pGAL1-10-NSE6*  *leu2::LEU2pRS305-NSE1-pGAL1-10-NSE2*  *his3::HIS3pRS303-NSE3-pGAL1-10-NSE4*  *ura3::URA3pRS306-SMC5-pGAL1-10-SMC6-TAP* |
| CB3623 | *MATa pep4∆::HPH top1∆::NAT*  *leu2::LEU2pRS305-NSE1-pGAL1-10*  *his3::HIS3pRS303-NSE3-pGAL1-10-NSE4*  *ura3::URA3pRS306-SMC5-pGAL1-10-SMC6-TAP* |
| CB3662 | *MATa pep4∆::HPH top1∆::NAT*  *trp1::TRP1pRS304-NSE5-pGAL1-10-NSE6*  *leu2::LEU2pRS305-NSE1-pGAL1-10-NSE2*  *his3::HIS3pRS303-NSE3-pGAL1-10-NSE4*  *ura3::URA3pRS306-smc5K75E-pGAL1-10-smc6K115E-TAP* |
| CB3663 | *MATa pep4∆::HPH top1∆::NAT*  *trp1::TRP1pRS304-NSE5-pGAL1-10-NSE6*  *leu2::LEU2pRS305-NSE1-pGAL1-10-NSE2*  *his3::HIS3pRS303-NSE3-pGAL1-10-NSE4*  *ura3::URA3pRS306-smc5E1015Q-pGAL1-10-smc6E1048Q-TAP* |
| CB3935 | *MATa pep4∆::HPH top1∆::NAT*  *trp1::TRP1pRS304-NSE5-pGAL1-10-NSE6*  *leu2::LEU2pRS305-NSE1-pGAL1-10-NSE2-6xHis-SNAP-KAN*  *his3::HIS3pRS303-NSE3-pGAL1-10-NSE4*  *ura3::URA3pRS306-SMC5-pGAL1-10-SMC6-TAP* |
| CB4005 | *MATa pep4∆::HPH top1∆::NAT*  *trp1::TRP1pRS304-NSE5-pGAL1-10-NSE6*  *leu2::LEU2pRS305-NSE1-pGAL1-10-NSE2*  *his3::HIS3pRS303-NSE3-pGAL1-10-NSE4-6xHis-SNAP-KAN*  *ura3::URA3pRS306-SMC5-pGAL1-10-SMC6-TAP* |
| CB4028 | *MATa pep4∆::HPH top1∆::NAT*  *leu2::LEU2pRS305-NSE1-pGAL1-10-NSE2*  *his3::HIS3pRS303-NSE3-pGAL1-10-NSE4-6xHis-SNAP-KAN*  *ura3::URA3pRS306-SMC5-pGAL1-10-SMC6-TAP* |
| CB4036 | *MATa pep4∆::HPH top1∆::NAT*  *trp1::TRP1pRS304-NSE5-TAP-URA3-pGAL1-10-NSE6* |
| CB4046 | *MATa pep4∆::HPH top1∆::NAT*  *trp1::TRP1pRS304-NSE5-6xHis-SNAP-KAN-pGAL1-10-NSE6*  *leu2::LEU2pRS305-NSE1-pGAL1-10-NSE2*  *his3::HIS3pRS303-NSE3-pGAL1-10-NSE4*  *ura3::URA3pRS306-SMC5-pGAL1-10-SMC6-TAP* |

**Table S2. Plasmid DNA used in this study.**

| *Name* | *Description* | *Source* |
| --- | --- | --- |
| pJF2 | pRS303::*CDT1-pGAL1-10-GAL4* | Gift from Prof. John Diffley^1^ |
| pJF3 | pRS304::*MCM4-pGAL1-10-MCM5* | Gift from Prof. John Diffley^1^ |
| pJF4 | pRS305::*MCM6-pGAL1-10-MCM7* | Gift from Prof. John Diffley^1^ |
| pJF5 | pRS306::*MCM2-pGAL1-10-MCM3* | Gift from Prof. John Diffley^1^ |
| CD373 | pRS306-*SMC5-pGAL1-10-SMC6-TAP* | This study |
| CD377 | pRS30-*NSE1-pGAL1-10-NSE2* | This study |
| CD380 | pRS304-*NSE5-pGAL1-10-NSE6* | This study |
| CD395 | pRS303-*NSE3-pGAL1-10-NSE4* | This study |
| CD402 | pFA6a-SNAP-KAN | This study |
| CD406 | pRS306-*smc5K75E-pGAL1-10-smc6K115E-TAP* | This study |
| CD420 | pRS306-*smc5E1015Q-pGAL1-10-smc6E1048Q-TAP* | This study |

**Captions for Supplementary Videos**

**Supplementary Video 1.** **Real-time imaging of DNA loop extrusion by Smc5/6 under side-way flow application.** The video corresponds to Fig. 1e.

**Supplementary Video 2.** **DNA loop extrusion by Smc5/6 in the absence of buffer flow.** The representative movie and the corresponding kymograph of a loop extrusion event are shown. The video corresponds to Fig. 1f-h.

**Supplementary Video 3.** **Side-flow visualization of DNA (cyan) loop extrusion by single fluorophore-labeled Smc5/6**. The Smc5/6 (red) binds on the DNA at ~7s and starts loop extrusion. The Smc5/6 stays at the stem of the loop before finally bleaching at ~151 s. The video relates to Fig. 2a.

**Supplementary Video 4.** **DNA loop extrusion by labeled Smc5/6 in the absence of buffer flow.** The kymograph and image sequences from DNA (top), labeled Smc5/6 (middle), and their merge (bottom). The video corresponds to Fig. 2e-g.

**Supplementary Video 5.** **Uni-directional translocation of a single Smc5/6 on DNA.** The time lapse video of DNA and Smc5/6 (right panel) and the corresponding kymograph (left panel) are shown. The video corresponds to Fig. 3a.

**Supplementary Video 6.** **A single translocating Smc5/6 complex dimerizes with another Smc5/6 whereupon loop extrusion is initiated.** The video corresponds to Fig. 3e,f.

**Supplementary Video 7.** **Nse5/6 enables high-salt resistant DNA binding of Smc5/6.** The video shows labeled Smc5/6 accumulated at the ends of DNA upon incubation of 1 hour, followed by a high salt wash. Upon introduction of high-salt buffer, Smc5/6 start diffusing away from the DNA ends and become re-distributed along the DNA. Gradual dissociation of Smc5/6 from the DNA was observed thereafter, with a small fraction of the protein remaining bound after 15 minutes of incubation in the high salt buffer. This video corresponds to Figure 4i,j.

**Supplementary Figure 1. Uncropped SDS-PAGE gel data from Extended Data Figures**. Gel source data for (a) ED Fig. 1a, (b) ED Fig. 1b, and (c) ED Fig. 1j.

**Supplementary Figure 2. Uncropped CBB stained SDS-PAGE gel data from Extended Data Figures**. Gel source data used for (a) ED Fig. 1d, (b) ED Fig. 1e, (c) ED Fig. 1f, (d) ED Fig. 1g, (e) ED Fig. 1h, (f) ED Fig. 1i, (g) ED Fig. 9a.
